# The *Achromobacter* type 3 secretion system drives pyroptosis and immunopathology via independent activation of NLRC4 and NLRP3 inflammasomes

**DOI:** 10.1016/j.celrep.2023.113012

**Published:** 2023-08-19

**Authors:** Keren Turton, Hannah J. Parks, Paulina Zarodkiewicz, Mohamad A. Hamad, Rachel Dwane, Georgiana Parau, Rebecca J. Ingram, Rebecca C. Coll, Clare E. Bryant, Miguel A. Valvano

**Affiliations:** 1 Wellcome-Wolfson Institute for Experimental Medicine, Queen’s University Belfast, Belfast BT9 7BL, UK; 2 Department of Medical Laboratory Sciences, University of Sharjah, Sharjah, United Arab Emirates; 3 Department of Veterinary Medicine, University of Cambridge, Cambridge CB3 0ES, UK; 4 Department of Medicine, Addenbrooke’s Hospital, Cambridge Biomedical Campus, Cambridge CB2 0QQ, UK

## Abstract

How the opportunistic Gram-negative pathogens of the genus *Achromobacter* interact with the innate immune system is poorly understood. Using three *Achromobacter* clinical isolates from two species, we show that the type 3 secretion system (T3SS) is required to induce cell death in human macrophages by inflammasome-dependent pyroptosis. Macrophages deficient in the inflammasome sensors NLRC4 or NLRP3 undergo pyroptosis upon bacterial internalization, but those deficient in both NLRC4 and NLRP3 do not, suggesting either sensor mediates pyroptosis in a T3SS-dependent manner. Detailed analysis of the intracellular trafficking of one isolate indicates that the intracellular bacteria reside in a late phagolysosome. Using an intranasal mouse infection model, we observe that *Achromobacter* damages lung structure and causes severe illness, contingent on a functional T3SS. Together, we demonstrate that *Achromobacter* species can survive phagocytosis by promoting macrophage cell death and inflammation by redundant mechanisms of pyroptosis induction in a T3SS-dependent manner.

## Graphical abstract

**Graphical abstract:**
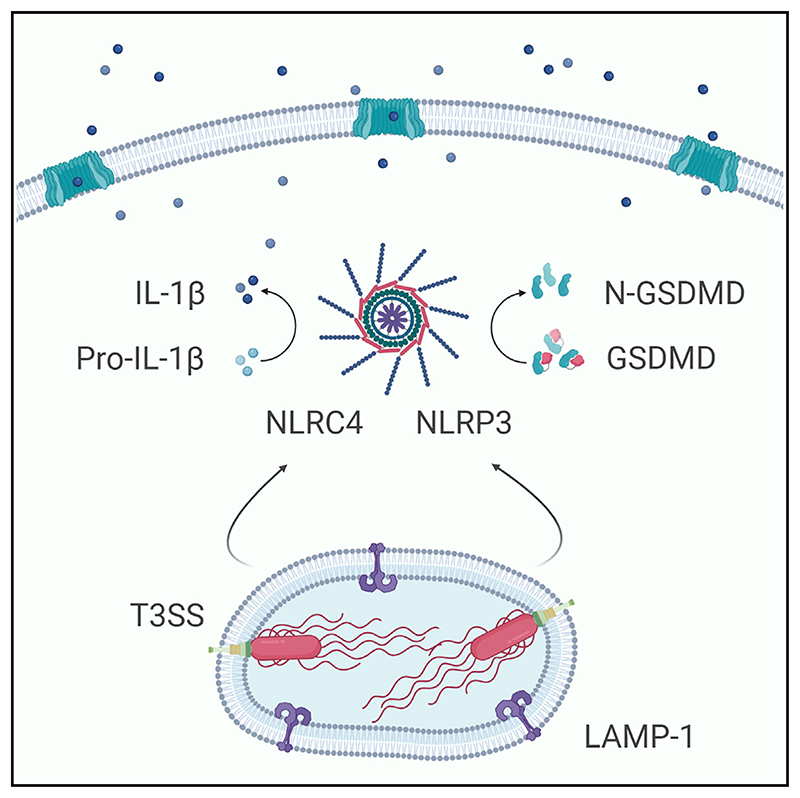

## Introduction

Species of the genus *Achromobacter*, primarily found in soil, are emerging opportunistic Gram-negative pathogens in immunocompromised individuals.^1^ In people with cystic fibrosis, the infection is recalcitrant, and re-colonization even after lung transplantation is common.^2^ Disease severity and symptoms are comparable with those of *Pseudomonas aeruginosa*, a common cystic fibrosis pathogen.^3^ Additionally, *Achromobacter* bacteremia^4–6^ and infections in patients with hematologic and solid organ malignancies, renal failure, and various immunodeficiencies have been reported.^1^ *Achromobacter* infections are difficult to treat because multi-drug resistance is common, and in cases of chronic infections the bacteria frequently acquire further resistance over time.^7,8^ This genus has received less attention than more ubiquitous lung pathogens, such as *Klebsiella* and *Pseudomonas*, but its increasing prevalence^1,9,10^ justifies further investigation. In addition to *A. xylosoxidans*, the primary focus in previous studies, several other *Achromobacter* species including *A. insuavis* and *A. ruhlandii* are clinically significant.^11^

*Achromobacter* species carry virulence and drug resistance genes that make them formidable during infection, particularly in immunocompromised individuals.^11,12^ Previous studies have reported the biofilm potential of *A. xylosoxidans* and the presence of an uncharacterized heat-stable cytotoxin.^9,12–15^ The *A. xylosoxidans* type 6 secretion system (T6SS) is involved in interactions with co-infecting lung pathogens and has been suggested to play a role in bacterial entry to alveolar epithelial cells.^16^ A type 3 secretion system (T3SS) with a similar gene organization to that in *Bordetella* has been identified in *Achromobacter* species.^17–20^ Little is known about the regulation of the *Achromobacter* T3SS and only one T3SS effector associated with cytotoxicity, the phospholipase AxoU, has been characterized in many strains of *A. xylosoxidans*,^21^ but it is absent in other species of the genus. Additionally, a repeats-in-toxin adhesion protein (RTX adhesin) contributes to the pathogenicity of *A. xylosoxidans* isolates by enhancing adhesion and internalization in macrophages.^22^ The pro-inflammatory potential of *Achromobacter* infections in people with cystic fibrosis has been clinically established,^23,24^ but how *Achromobacter* species interact with the innate immune system to elicit inflammation remains unknown. This work aims to unravel macrophage-.*Achromobacter* interactions, since macrophages are one of the front-line defenders of the immune system. We investigated macrophage cell death after *Achromobacter* infection using the *A. insuavis* isolate AC047 and two clinical isolates of *A. xylosoxidans*. AC047 is cytotoxic to human macrophages despite lacking the characterized virulence factors AxoU and RTX adhesin, suggesting that macrophage cell death mediated by *Achromobacter* species is multifactorial. In this study, we created a set of mutations in *A. insuavis* AC047 and two *A. xylosoxidans* isolates to delineate the role of T3SS components in infection and inflammation using human macrophage cell models and a mouse model of respiratory infection.

Pyroptosis is a form of programmed cell death that results in the activation of inflammatory caspases leading to the cleavage of the pore forming protein gasdermin D and the release pro-inflammatory cytokines.^25,26^ The activation of pyroptotic programming can be induced by several Gram-positive and Gram-negative pathogens, as well as endogenous signals, and contributes to the recruitment of immune cells. In this work, we show that human macrophages undergo pyroptosis when infected by clinical isolates of *Achromobacter* species and that this process is T3SS dependent. We used a panel of human macrophage cell lines deficient in specific inflammasome components to assess the contributions of Nod-like receptor (NLR) family CARD domain-containing protein 4 (NLRC4) and NLR family pyrin domain containing 3 (NLRP3), which can sense pathogen-associated molecular patterns and danger signals in the cytosol.^25^ We demonstrate that the NLRC4 and NLRP3 inflam-masome sensors are each sufficient for pyroptosis induction, but neither is absolutely required. Additionally, we used an *A. xylosoxidans* strain conferring low cytotoxicity to study the intracellular trafficking of the bacterium in macrophages, revealing that intracellular survival of *Achromobacter* occurs in a late phagosome and is T3SS independent. The T3SS was also required to elicit lung inflammation and tissue damage in a mouse acute infection model, and death in the *Galleria mellonella* wax moth infection model, indicating that this system playsasignificant role in the pathogenicity of *Achromobacter* species *in vivo*.

## Results

### The T3SS of *Achromobacter* species is necessary to induce macrophage cell death

**Figure 1:**
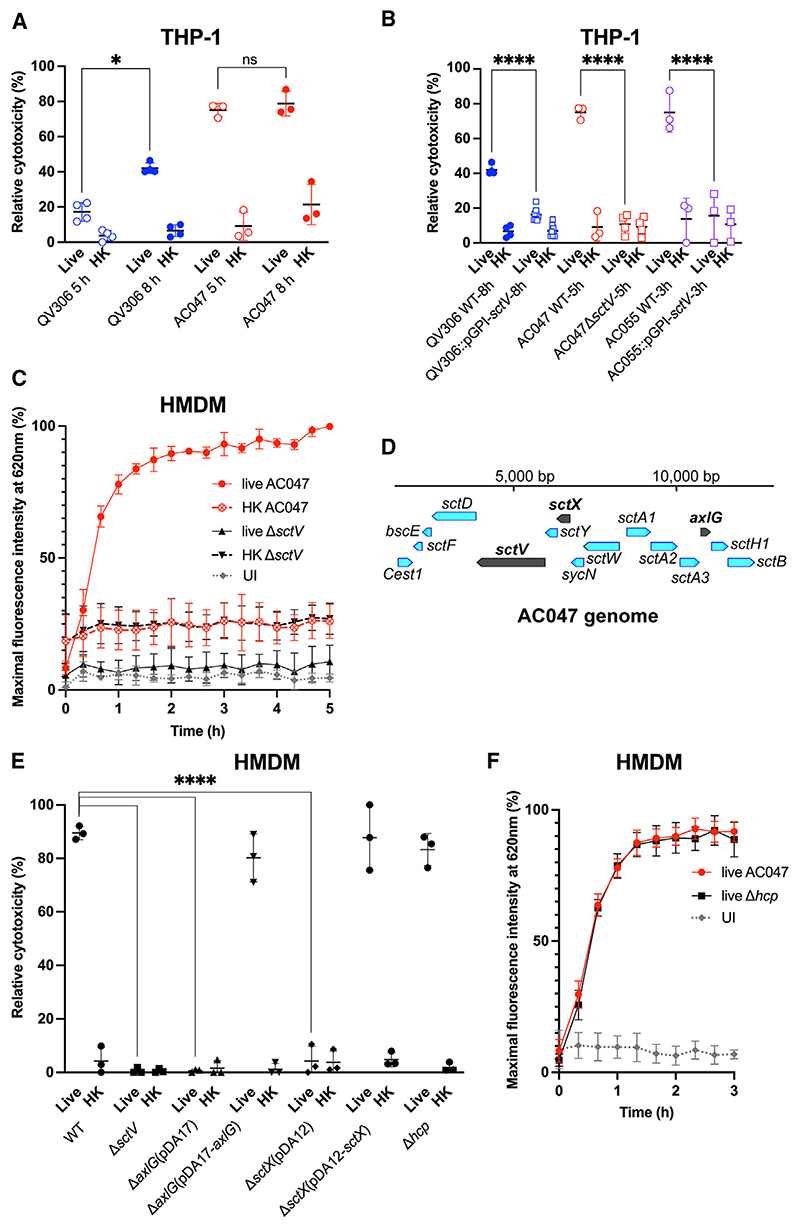
The T3SS is required for induction macrophage cell death (A) LDH release of THP-1 cells infected with live and heat-killed (HK) bacteria (paired t tests). (B) LDH release of THP-1 cells infected with live and *sctV* mutants (paired t tests). (C) PI uptake time-course assay of HMDM infection with strain AC047 and Δ*sctV* mutant. (D) Partial schematic of AC047 T3SS operon indicating the deleted genes, generated using Benchling. (E) LDH release of HMDM infected with live and heat-killed (HK) AC047 and its Δ*sctX* and Δ*axlG* mutants at 5 h p.i. (two-way ANOVA). (F) PI assay of WT and Δ*hcp* AC047 infecting HMDM. Multiplicity of infection (MOI) 20, error bars SD. Data are represented as mean ± SD from at least three biological replicates with four technical replicas each. UI, uninfected. MOI 20; ****p < 0.0001; *p < 0.05.

The ability of the *Achromobacter* clinical isolates *A. insuavis* AC047 and *A. xylosoxidans* QV306 to induce cell death in macrophages was assessed by monitoring lactate dehydrogenase (LDH) release at 5 and 8 h after infection. Both strains induced cell death in THP-1 macrophages, albeit on different timescales (Figure 1A). Cytotoxicity required viable bacteria; the cytotoxicity induced by heat-killed bacteria in control experiments was negligible. The results also suggested the degree of cytotoxicity is strain dependent, possible because of differences in bacterial uptake by macrophages, but since we could not kill off extracellular bacteria, we could not establish whether this was significant. To investigate whether cytotoxicity by *Achromobacter* species is associated with the T3SS, we constructed mutants in AC047, QV306, and in another *A. xylosoxidans* isolate, AC055, by inactivating the *sctV* gene encoding the baseplate protein of the T3SS. This gene was selected for our initial mutagenesis experiments since the baseplate protein in *Salmonella*^27^ and *Yersinia*^28^ T3SSs is critical for secretion and needle assembly. We generated an unmarked *sctV* deletion in AC047 (Δ*sctV*) and insertional *sctV* mutants (pGPI-*sctV*) in *A. xylosoxidans* QV306 and AC055. All three mutants failed to cause macrophage cell death (Figure 1B), indicating that the *Achromobacter* T3SS is required for cell death induction in infected THP-1 macrophages.

We also examined primary human monocyte-derived macrophages (HMDMs) infected with AC047 by the LDH release endpoint assay and a propidium iodide (PI) uptake assay, which allowed us to follow the time course of cytotoxicity over 5 h. As observed for LDH release in THP-1 macrophages, wildtype but not AC047Δ*sctV*, induced rapid cytotoxicity in HMDMs (Figure 1C), reaching nearly 80% of its peak within 1 h, in contrast with heat-killed or Δ*sctV* bacteria, which did not induce significant PI uptake. Irrespective of the presence of the *sctV* gene, macrophages exposed to heat-killed bacteria showed basal PI readings around 10% higher than uninfected macrophages, and macrophages infected with live Δ*sctV* (Figure 1C). We attributed these slightly higher values to PI fluorescence arising from the bacterial DNA in the heat-killed bacterial controls, since PI cannot access live bacterial cells, which was confirmed by performing the PI assay on heat-killed bacteria (Figure S1A).

To demonstrate that the T3SS was necessary for cytotoxicity in HMDMs, we constructed a complementation plasmid by expressing SctV as a C-terminal FLAG fusion protein. The complemented AC047Δ*sctV* strain induced cytotoxicity in HMDMs but at lower levels than the wildtype bacteria (Figure S1B). Since the SctV protein was expressed, as demonstrated by western blot (Figure S1C), the partial complementation could be attributed to polar effects of the *sctV* deletion or partial protein instability caused by the FLAG epitope. Therefore, we constructed additional gene deletions in *sctX* (a homologue of *yscX*, 408 bp) and *axlG* (312 bp) (see schematic indicating the deleted genes in the AC047 T3SS cluster in Figure 1D). In *Yersinia*, YscX is essential for early secretion from the T3SS.^29,30^ The AxlG protein is a homologue of the chaperone protein Btc22, which is required for the secretion of the needle protein Bsp22 (SctA) in the *Bordetella* T3SS.^31^ Analysis of the T3SS cluster by cblaster^32^ in 208 *Achromobacter* genomes revealed that *axlG* was immediately adjacent to *sctA* in 98% of them (Figure 1D). The standard nomenclature was used to refer to the T3SS genes whenever possible,^33^ but *axlG* is not a core component of the T3SS in other bacterial species. We, therefore, named this gene according to the label system created by Pickrum et al.^21^ for the equivalent gene in their *Achromobacter* strain. Deletion of *sctX* and *axlG* genes completely abrogated cytotoxicity, but complementation with the respective parental genes restored it (Figure 1E). *Achromobacter* species have also a T6SS, which plays a role in inter-bacterial competition and in the infection of respiratory epithelial cells.^16^ To rule out the T6SS as a contributor to macrophage cell death, we generated a mutant lacking Hcp, an essential protein component of the T6SS apparatus (there is only one copy of the *hcp* gene in AC047). The disruption of *hcp* did not affect LDH release or PI uptake (Figures 1E and 1F), showing that infection-induced cell death in macrophages is T6SS independent. This is also supported by the lack of residual cytotoxicity in macrophages infected with the T3SS-defective mutants (Figure 1E) where the T6SS remained intact.

### Cell death is contact dependent and enhanced by bacterial internalization

Having established that the T3SS is required for macrophage cell death, we next investigated if this process required bacteria-cell contact, internalization, or both. Because *Achromobacter* species are multidrug resistant, we could not perform a classical gentamicin assay to kill extracellular bacteria during infection. Various other combinations of antibiotics were trialed but ultimately deemed unsatisfactory since the high antibiotic thresholds required led to macrophage cytotoxicity or the killing of intracellular bacteria, as infection-induced cytotoxicity culminated in membrane disruption of host macrophages. Although we washed bacteria repeatedly before suspension in RPMI to remove effectors in the culture medium, it was possible that bacteria could secrete more effectors during infection. To circumnavigate these issues, we used transwells^34^ to demonstrate whether bacteria-cell contact was required to induce cytotoxicity. This experiment demonstrated that *Achromobacter* must be in contact with macrophages to cause cytotoxicity, as LDH release is abrogated in the transwells (Figure S2A). We next investigated whether internalization was a prerequisite for cytotoxicity by pretreating macrophages with 5 μg/mL cytochalasin D (CD), which inhibits phagocytosis.^35^ In HMDMs, CD delayed the onset of cytotoxicity as measured by the PI permeability assay (Figure S2B). Fluorescence did not increase in the uninfected CD-treated control or in macrophages exposed to the DMSO vehicle. We also examined the effect of CD in THP-1 macrophages infected with QV306, AC047, and AC055 using confocal microscopy by following bacteria expressing the mCherry fluorescent protein. At 5 h post infection (p.i.), we observed no internalized *Achromobacter* species inside CD-treated THP-1 macrophages (Figure S2C). We also observed that the *A. xylosoxidans* QV306 and AC055 bacterial cells adhered to the surface of CD-treated cells, while the *A. insuavis* AC047 bacteria did not (Figure S2C). We speculate these differences reflect the presence of the RTX adhesin,^22^ which is encoded in *A. xylosoxidans* QV306 and AC055, but absent in the *A. insuavis* AC047 genome, and can promote cytotoxicity upon bacterial contact with the surface of target cells. Together, these experiments demonstrate that macrophage cell death requires both bacteria-cell contact and internalization.

### *Achromobacter* species reside in a late phagosome compartment in a T3SS-independent manner

**Figure 2:**
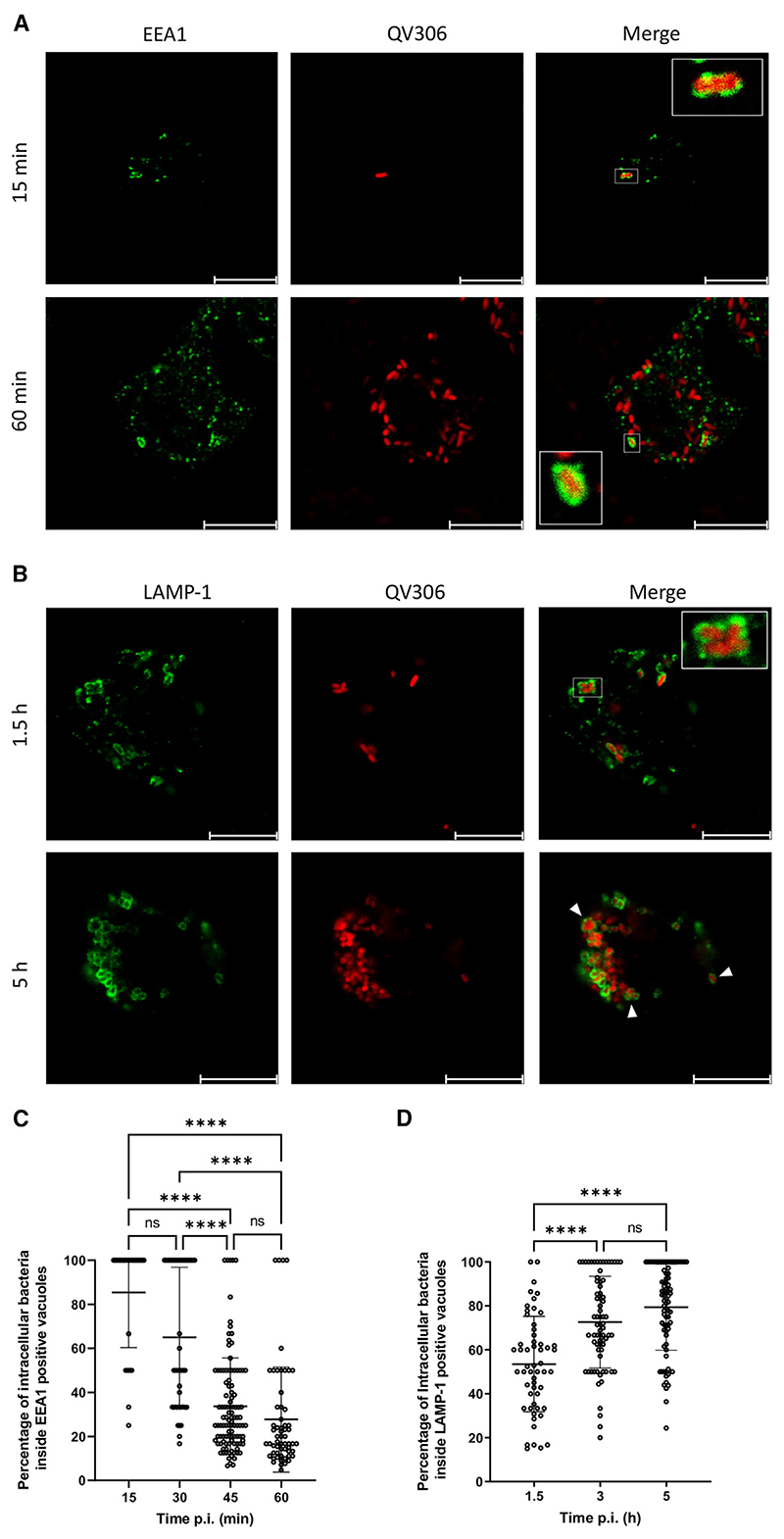
*A. xylosoxidans* QV306 infecting THP-1 macrophages resides in a vacuole that resembles a late endosomal compartment (A) Live QV306 bacteria infecting macrophages at 15 and 60 min p.i. are in membrane vacuoles colocalizing with EEA1. (B) Live QV306 bacteria infecting macrophages at 1.5 and 5 h p.i. co-localize with LAMP-1. Images taken with ×63 magnification on a Leica SP8 confocal microscope. Multiplicity of infection, 80. Scale bar, 10 μm. (C) The percentage of intracellular bacteria in EEA1-positive vacuoles was assessed by counting in at least 40–100 macrophages. (D) The percentage of intracellular bacteria present in LAMP-1-positive vacuoles was assessed by counting in at least 80 macrophages. ****p < 0.0001 by Kruskal-Wallis test. Data are represented as mean ± SD from at least three biological replicates. NS, not significant.

**Figure 3:**
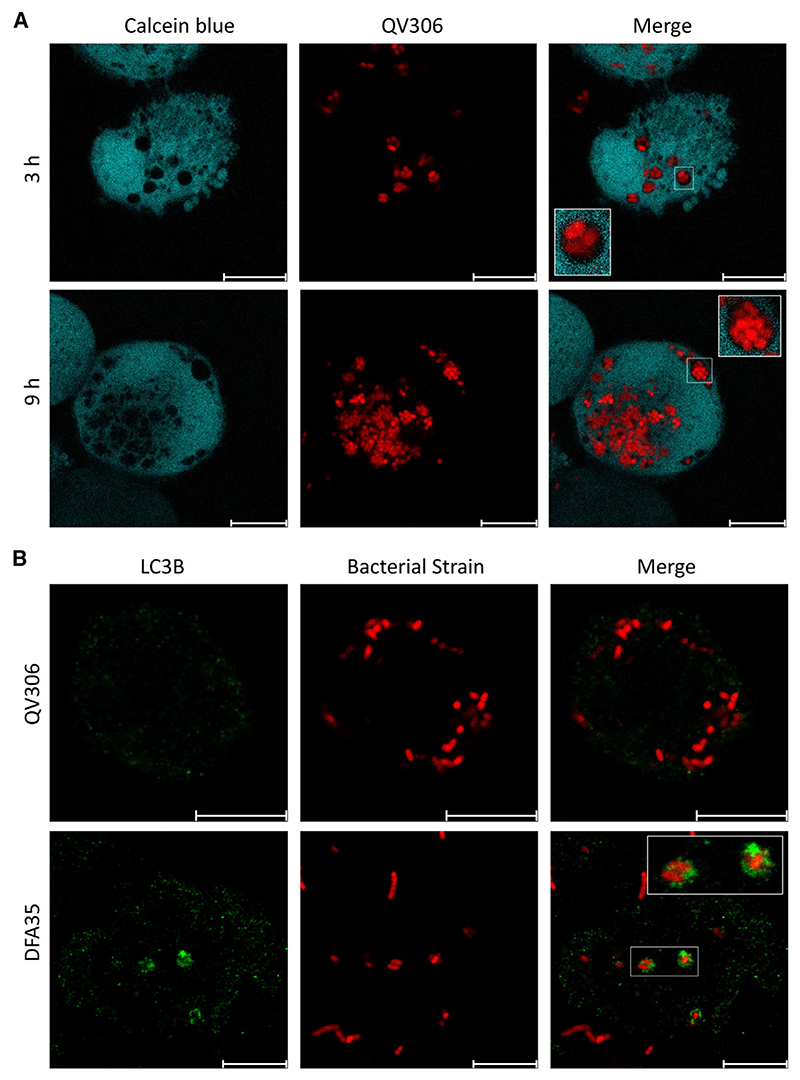
QV306 localized to intracellular vacuoles that do not recruit the early autophagy marker LC3B (A) THP-1 macrophages 3 and 9 h p.i. with QV306. Cells were stained with Calcein blue (pseudocolored as cyan) and processed for live imaging on a Leica Stellaris-5 confocal microscope (original magnification × 100). (B) Infected THP-1 macrophages 3 h p.i. were imaged by immunofluorescence using an anti-LC3B polyclonal antibody. (Top) The QV306 AcVs do not colocalize with the autophagosome marker LC3B. (Bottom) (control) Vacuoles containing *Burkholderia cenocepacia* isolate DFA35 colocalize with LC3B. Images taken with ×63 magnification on Leica SP8 confocal microscope. Multiplicity of infection, 80. Scale bar, 10 μm.

*A. xylosoxidans* has been reported to survive intracellularly without significant replication, exiting infected cells upon cytotoxic membrane rupture.^22^ To understand the intracellular trafficking and survival strategy of *Achromobacter* species after phagocytosis, we investigated the maturation of the *Achromobacter*-containing vacuole (AcV) in THP-1 macrophages infected with the less acutely cytotoxic *A. xylosoxidans* strain QV306 expressing mCherry. Strains AC047 and AC055 rapidly destroy the macrophages and escape the phagosome and were thus less useful than QV306 for long-term visualization. After phagocytosis, the AcV transiently acquired the early endosomal antigen 1 (EEA1) marker, but its association with this marker declined over 1 h p.i. (Figure 2A). In contrast, the AcV associated over time with the late endosome/phagolysosome marker LAMP-1 (Figure 2B). Image quantifications (Figures 2C and 2D) demonstrate that, while the association of the AcVs with EEA1 decreases over time, the association with LAMP-1 increases and virtually all AcVs are LAMP-1-positive after 3 h p.i. Similar kinetics of the AcV localizing with LAMP-1 was observed in HMDMs (Figure S3A). Using Calcein blue staining of the cytoplasm, a dye that does not enter membrane vacuoles, we confirmed that QV306 bacteria are both internalized and confined to a membrane compartment (Figure 3A). *A. insuavis* AC047 also localized in a LAMP-1-rich AcV within THP-1 macrophages early after infection (Figure S3B), although cytotoxicity compounded to the difficulty to kill extracellular bacteria precluded late-stage analyses.

Other pathogens associated with cystic fibrosis, such as *Burkholderia cenocepacia*, accumulate the autophagy marker LC3B early after phagocytosis.^36^ In contrast with THP-1 macrophages infected with *B. cenocepacia*, which were used as control to show the presence of LC3B associated with the bacteria-containing vacuole at 3 h p.i., the AcV was not associated with LC3B (Figure 3B), indicating that the AcV is not an autophagic vacuole. Dextran fluorescein is a fluid phase marker that demonstrates co-localization in the phagolysosome, where ingested bacteria are degraded. At 3 and 5 h p.i., the AcV co-localizes with dextran fluorescein (Figure S4). This supports our LAMP-1 co-localization data (Figure 2B), corroborating that the bacteria are in a late phagosome compartment. We also investigated whether the AcV matures to become acidic by evaluating the accumulation of Lysotracker Green DND. The results show that some but not all AcVs seem to be acidic compartments (Figure S5). This heterogeneity could be caused by the presence of extracellular bacteria that are differentially internalized over time during the experiment, since we cannot effectively kill either the residual extracellular bacteria at the onset of the experiment or re-uptake of intracellular bacteria released after macrophage cell lysis. We also infected HMDMs with mCherry-labelled AC047Δ*sctV* to investigate whether the T3SS is required for intracellular survival. The results show that the mutant can be internalized in the absence of a T3SS and accumulates in a LAMP-1-positive vacuole containing intact bacterial cells for at least 24 h p.i. (Figure S6). Together, our data indicate that *Achromobacter* strains can reside in a late phagosome compartment and this process is T3SS independent.

### *Achromobacter*-induced macrophage cell death occurs by T3SS-dependent pyroptosis and requires NLRP3 and NLRC4 inflammasome sensors

**Figure 4:**
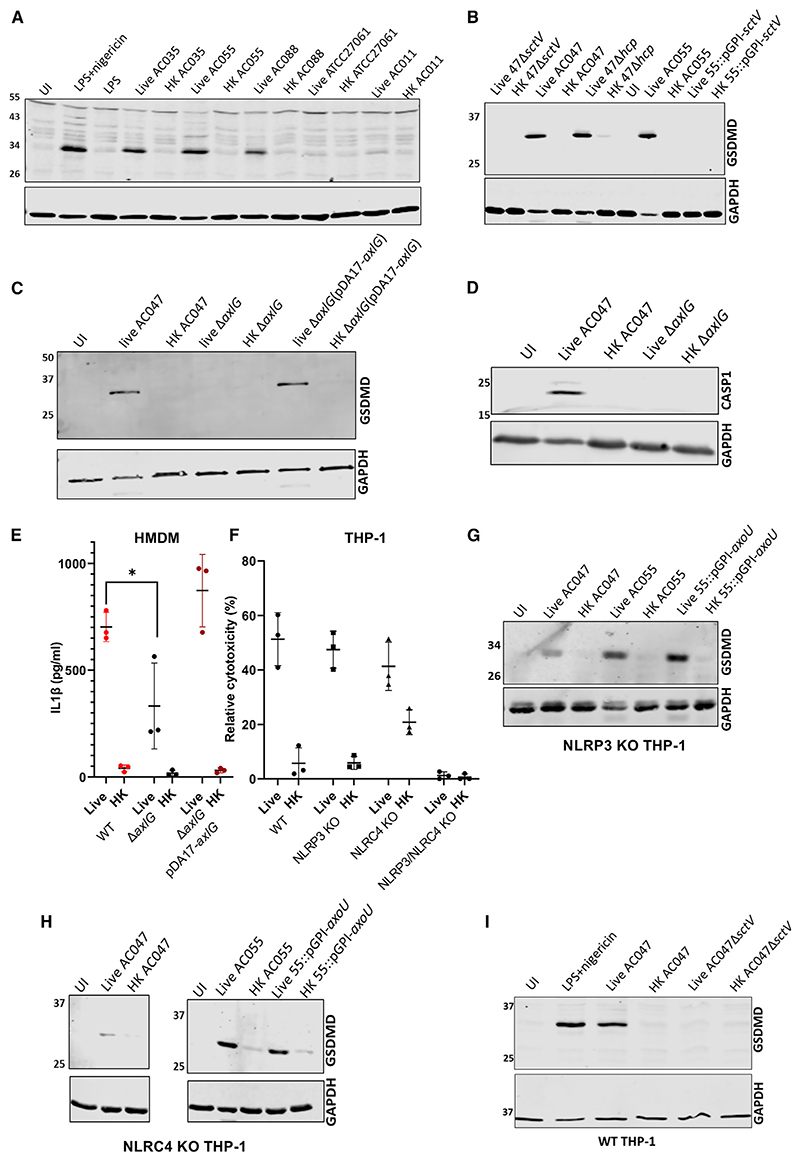
The T3SS is required for NLRC4- and NLRP3-dependent pyroptosis in macrophages All infections were carried out with a multiplicity of infection of 20 and followed for 5 h. (A) Gasdermin-D cleavage in THP-1 infected with live and heat-killed (HK) *Achromobacter* sp. using antibody RRID:AB_2800210. (B and C) Gasdermin-D cleavage in HMDM infected with live and HK AC047, AC055, and mutants. Antibody (RRID:AB_2799099) detects N terminus of cleaved GSDMD. (D) Caspase-1 cleavage in HMDM infected with live, HK AC047, and mutant. Antibody (CST #4199) detects cleaved caspase 1. (E) ELISA of IL-1β in supernatants of infected HMDM, 3 h p.i. (t test). (F-I) LDH release of infected KO THP-1 lines (two-way ANOVA). Gasdermin-D and GAPDH immunoblots of NLRP3 KO (G), NLRC4 KO (H), and WT (I) THP-1 infected with live and HK bacteria. Data are represented as mean ± SD from at least three biological replicates. *p < 0.05; ***p < 0.001.

The innate immune system can recognize pathogen-associated molecular patterns such as T3SS components that trigger pyroptosis.^25^ To establish whether macrophage cell death is caused by pyroptosis, we examined cell lysates from THP-1 macrophages infected with several clinical isolates of *Achromobacter* for gasdermin-D (GSDMD) cleavage, a proxy for a pro-inflammatory response leading to the formation of membrane pores and release of mature interleukin (IL)-1β and IL-18 into the external milieu.^37^ GSDMD cleavage, as assessed by western blot detection of its cleaved N-terminal domain, was observed in infected macrophages with all live (but not heat-killed) strains containing T3SS (AC035, AC055, and AC088) to a level comparable with GSDMD cleavage triggered by NLRP3 activation, which is mediated by treatment of macrophages with lipopolysaccharide (LPS) and nigericin (Figure 4A). The *A. xylosoxidans* isolates AC055 and AC088, and the *Achromobacter* species cluster-II isolate AC035, but not the *A. xylosoxidans* AC011 and ATCC27061 (type strain) isolates, contained T3SS systems based on genomic DNA sequencing and PCR amplifications (Figure S7A). An intact T3SS was required for pyroptosis induction by AC047, AC055, and QV306, as GSDMD cleavage was absent in HMDMs infected with their corresponding *sctV* mutants (Figures 4B and S7B), but unaffected in the T6SS (Δ*hcp*) mutant. As expected, complementing the deleted *axlG* gene in the AC047Δ*axlG* mutant restored GSDMD cleavage (Figure 4C). Infection with live AC047 also resulted in caspase-1 cleavage and IL-1ß release, an effect not seen in the T3SS-deficient mutant (Figures 4D and 4E). When complemented, AC047Δ*axlG* released IL-1β levels comparable with wildtype. Treatment with VX-765, a caspase-1/4 inhibitor,^38^ delayed the onset of cytotoxicity in infected HMDMs as measured by PI uptake (Figure S7C), providing further evidence of caspase-1-mediated gasdermin-D cleavage. Collectively, these experiments indicate that a functional T3SS is required to induce pyroptosis in THP-1 macrophages and HMDMs.

Several inflammasome sensors, including NLRC4 and NLRP3, can detect intracellular Gram-negative pathogens.^25^ NLR family of apoptosis inhibitory proteins (NAIPs) are innate immune sensors^39^ that can co-assemble with NLRC4 upon detection of T3SS components^40^; in some instances, depending on the macrophage model, NLRP3 can also be involved.^41^ We investigated whether NLRC4 or NLRP3 participate in detecting *Achromobacter* infection using a set of NLRP3, NLRC4, and NLRP3/NLRC4 knockout (KO) THP-1 cells that were previously well characterized^41,42^ and matched WT THP-1 control cells from the same source as the KO cell lines. The results show that NLRC4 and NLRP3 sensors were each sufficient, but neither was solely necessary for cytotoxicity (Figure 4F), which was corroborated by GSDMD immunoblotting data (Figures 4G and 4H) and IL-1β ELISAs detecting the released cytokine in cell supernatants (Figure S8A), indicating that pyroptosis still occurred in each of the single KO cell lines as in WT THP-1 (Figure 4I). Flagellin is involved in both NLRC4- and NLRP3-dependent pyroptosis induction in *Salmonella* infection.^41^ We constructed a *fliC* mutant in AC047 to determine if the absence of *fliC* affected cytotoxicity in AC047 infection of NLRC4 KO or NLRP3 KO THP-1. There was no significant difference between WT and Δ*fliC* cytotoxicity (Figure S8B) in either KO cell line, suggesting an alternate mechanism for inflammasome activation that does not involve flagellin. The heightened basal reactivity of NLRC4 KO cells to HK wildtype bacteria (Figure 4F) and HK Δ*fliC* mutant is paralleled by higher levels of IL-1β release from NLRC4 KO cells treated with HK wildtype and Δ*sctV* mutant compared with the other KO and the WT THP-1 cells (Figure S8A) and could be explained by a possible dysregulation of NLRP3-driven responses in the absence of NAIP/NLRC4^43,44^ or direct caspase 4-mediated inflammasome activation.^45^

### Effector AxoU is cytotoxic independent of NLRC4 and NLRP3 and is not required for pyroptosis

**Figure 5:**
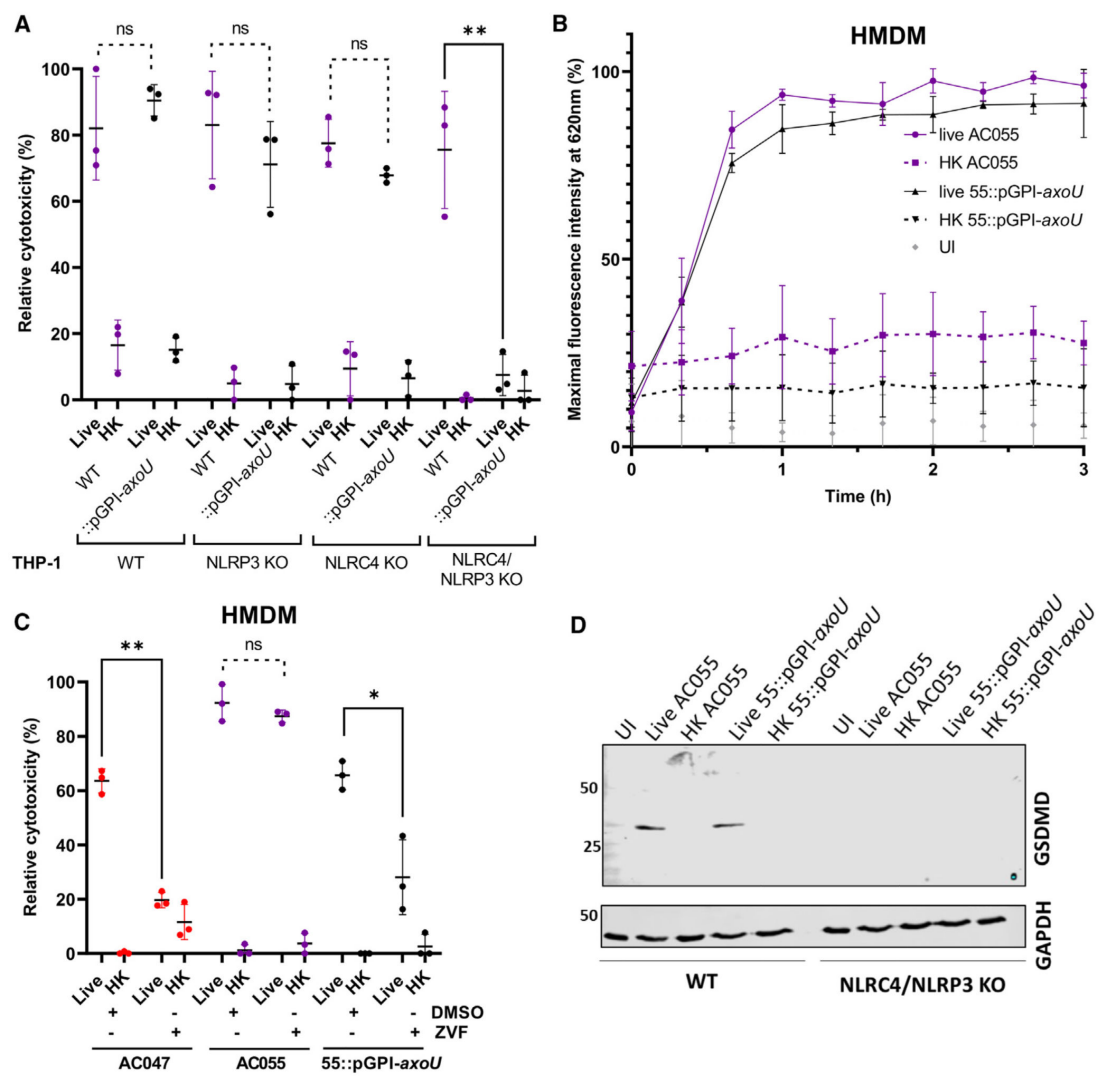
AxoU is not necessary for pyroptosis All infections were carried out with a multiplicity of infection of 20 for at least 5 h unless indicated otherwise. (A) LDH assays of AC055 and disrupted AxoU mutant infecting WT and THP-1 KO cells. (B) PI assay cytotoxicity time-course of HMDM infected with AC055 or with AC055pGPI-*axoU*. (C) LDH assay of HMDM cells infected with live/HK bacteria and treated with 10 μg/mL Z-VAD-FMK (ZVF) oran equivalent volume of dimethylsulfoxide (DMSO); 3 h p.i. (D) AC055pGPI-*axoU* induces GSDMD cleavage in WTTHP-1, but neither wildtype AC055 nor AC055pGPI-*axoU* induce GSDMD cleavage in dKO THP-1. Data are represented as mean ± SD from at least three biological replicates. **p < 0.005; *p < 0.05 by t test. ns, not significant.

Previous work in *A. xylosoxidans* identified AxoU, a homolog of the *P*. *aeruginosa* T3SS-secreted ExoU phospholipase A_2_, which induced cell death in macrophages when expressed in *P. aeruginosa* and delivered by the *P. aeruginosa’s* T3SS.^21^ To investigate the role of AxoU in cell death, we generated mutants of AC055 with disrupted *axoU* and *sctV* genes. Disrupting *axoU* abrogated macrophage cell death (Figure 5A). In contrast, disrupting *sctV* prevented cell death in HMDMs and THP-1 macrophages (Figure 1B) but disrupting *axoU* alone did not (Figure 5B), demonstrating that AxoU is not the only cytotoxic T3SS effector. Wildtype AC055, unlike the AC055 *axoU* mutant, was cytotoxic to NLRC4/NLRP3 KO THP-1 (Figure 5A), suggesting that *axoU*-mediated cytotoxicity is independent of these sensors. Similarly, treatment with pan-caspase inhibitor Z-Val-Ala-Asp(OMe)-fluoromethylketone (Z-VAD-FMK) abrogated cell death induction by the AC055 mutant lacking AxoU (Figure 5C), but not by wildtype AC055 with a functional AxoU. Z-VAD-FMK inhibits both pyroptosis and apoptosis, suggesting that AxoU induces cytotoxicity independently of both these pathways. We also examined whether AC055pGPI-AxoU could still elicit GSDMD cleavage. In NLRC4 KO, NLRP3 KO (Figures 3F and 3G), and WT THP-1 cells (Figure 5D), GSDMD cleavage occurred even when the *axoU* gene was disrupted, while in the NLRC4/NLRP3 KO THP-1 no pyroptosis occurred upon infection with wildtype or AC055pGPI-*axoU* (Figure 5D). QV306 also has an *axoU* gene, but since it is less cytotoxic than AC055, its disruption did not markedly alter the phenotype. The *axoU* gene is present in many *A. xylosoxidans* strains, but absent in the *A. insuavis* AC047, which can induce pyroptosis. AC055pGPI-AxoU mirrors the phenotypes observed for AC047 (Figures 3E, 5B, and 5C). Together, these data indicate AxoU is not required for pyroptosis induction.

### *Achromobacter* T3SS is required for virulence and inflammation *in vivo*

**Figure 6:**
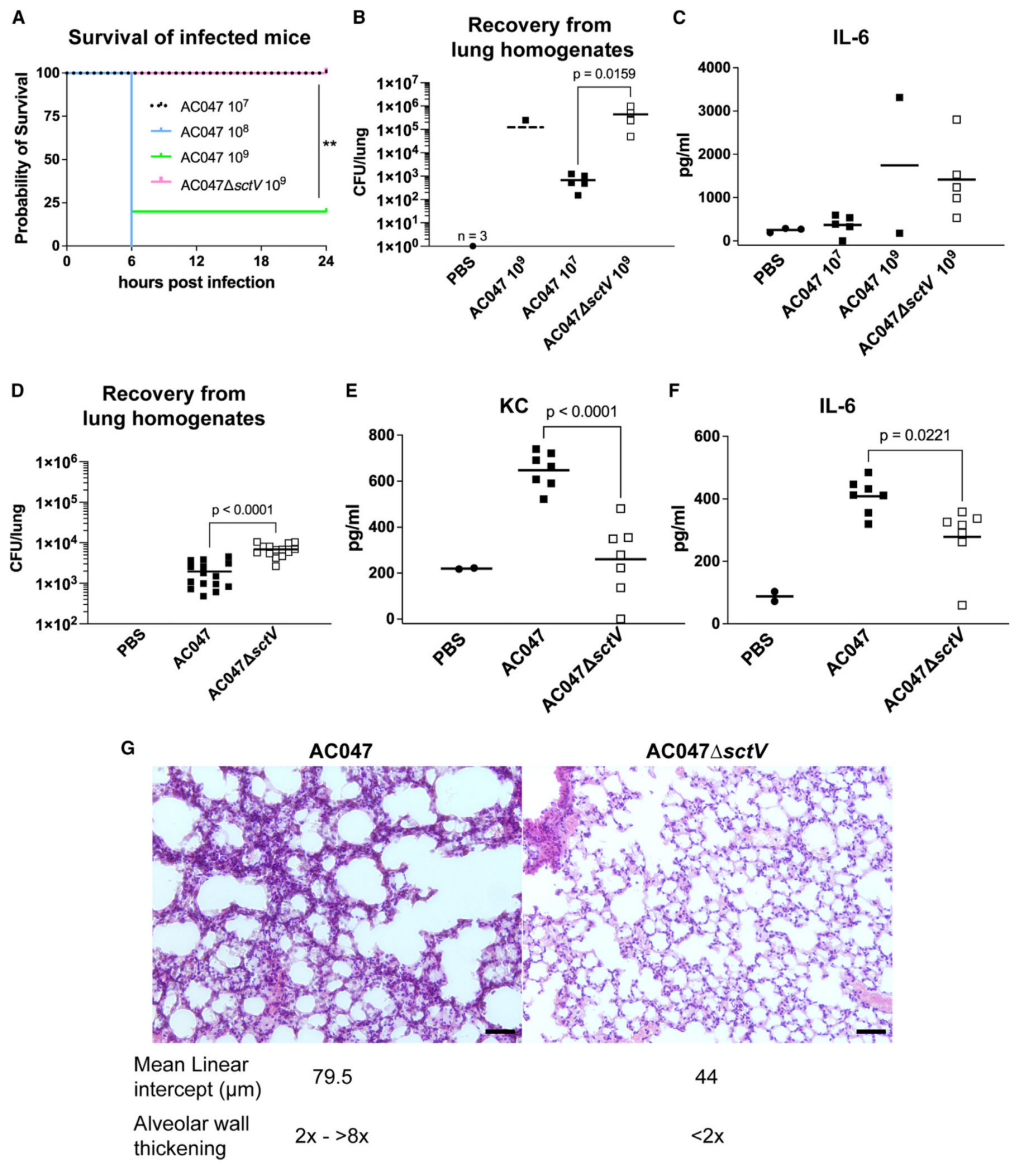
AC047Δ*sctV* is less virulent, causes lower inflammatory response, and does not cause acute lung damage in comparison toAC047 in mice (A-C) Mice infected intranasally with 10^9^ and 10^8^ of AC047 experience decreased survival within 6 h p.i. In comparison, mice infected with 10^9^ AC047Δ*sctV* survive 24 h without developing any symptoms but produce an inflammatory response shown by increased IL-6. (D-F) Mice infected intranasally with 10^7^ AC047Δ*sctV* have lower recoverable bacterial load from lung homogenates and show a lower inflammatory response shown by IL-6 and keratinocyte chemoattractant (KC) levels. (CFU is a sum of two experiments). (G) AC047Δ*sctV* infected mice (left) show smaller alveolar destruction and no wall thickening, in comparison to AC047 infected mice (right). Scale bar, 50 μm. *p < 0.05; **p < 0.005; ***p < 0.001 by t test (ELISAs) or Mantel-Cox test (survival).

We also investigated the pathogenic role of the T3SS in two *in vivo* models. First, we compared AC047 wildtype and AC047-Δ*sctV* in the *Galleria mellonella* wax moth model, which is commonly used to rapidly assess bacterial virulence.^46^ Larvae injected with wildtype AC047 died within 5 days p.i., whereas survival of larvae injected with AC047Δ*sctV* was comparable with the PBS-injected control (Figure S9), demonstrating that that a functional T3SS is required for *Achromobacter* virulence *in vivo*. Next, we assessed whether the T3SS is required for *Achromobacter* to establish a respiratory infection in a mouse model of acute lung infection by inoculating C57BL/6 mice intranasally. Mice administered 10^9^ and 10^8^ colony-forming units (CFU) wildtype AC047 reached a humane endpoint requiring to be euthanized within 6 h after inoculation, whereas mice treated with the same dose of AC047Δ*sctV* showed no symptoms during the 24 h p.i. (Figure 6A). These results suggested that loss of the T33S function causes attenuation of the AC047 pathogenicity. To better compare the pathogenicity of AC047 and AC047Δ*sctV*, we performed additional experiments inoculating the mice with 10^7^ CFU, which allowed us to recover bacteria from the lungs while no symptoms of illness appeared over 24 h (Figure 6B). Quantification of recovered bacteria in the lungs of these animals indicated a reduction in CFU/lung with respect to the initial inoculum, suggesting bacteria can be cleared over time. However, upon examination an inflammatory response was observed, as revealed by increased IL-6 production in the lung homogenates (Figure 6C). Subsequent infection experiments with 10^7^ CFU wild-type AC047 and AC047Δ*sctV* showed that a lack of T3SS results in slower bacterial clearance from the lungs and significantly lower inflammatory responses in comparison with wildtype, as seen by reduced IL-6 and keratinocyte chemoattractant levels (Figures 6D–6F). Supporting these observations, lung histology demonstrated that the AC047Δ*sctV*-infected mice had no obvious signs of inflammation or lung damage (Figure 6G). However, wildtype-infected mice showed a more than 4-fold increase in alveolar septa thickness and neutrophils in the interstitial space, which are parameters for assessment of acute lung injury.^39^ We observed more alveolar wall damage (mean linear intercept) in the AC047-infected mice in comparison with the AC047Δ*sctV*-infected mice. Similar histopathology as seen with the wild-type AC047 has been recently reported for *A. xylosoxidans* clinical isolates using an intratracheal infection model.^47^ Together, our data suggest that the T3SS contributes to eliciting an inflammatory response in the lung that causes significant tissue damage and results in more rapid bacterial clearance.

## Discussion

This study reveals how clinical isolates of *A. insuavis* and *A. xylosoxidans* exploit the T3SS to interact with human macrophages in a pro-inflammatory fashion resulting in pyroptosis. The T3SS, a bacterial nanomachine that delivers protein effectors into eukaryotic cells, is a crucial mediator of host-microbe interactions for many Gram-negative pathogens.^48^ We established that T3SS-defective *Achromobacter* mutants lost the ability to induce pyroptosis in macrophages, which required bacteria-macrophage contact and bacterial internalization. When evaluating other clinical isolates in our collection, including the type-strain for the genus *Achromobacter* (*A. xylosoxidans* ATCC27061, isolated from purulent otitis^49^), it became clear that those capable of killing macrophages also had T3SS operons, suggesting T3SS-mediated pyroptosis could be of broad clinical relevance.

The high-level multidrug antibiotic resistance of *Achromobacter* species, particularly of *A. xylosoxidans*, hampers the genetic manipulation of this genus because of the lack of appropriate drug selection markers. We discovered that the *A. insuavis* clinical strain AC047 was genetically tractable because it lacked tetracycline resistance, commonly found in *A. xylosoxidans*,^11^ which enabled us to use a two-step recombinational approach leading to the generation of markerless deletion mutants by adapting a mutagenesis protocol previously developed for *Burkholderia*.^50^ This approach allowed us to create a set of deletion mutants that helped to establish unequivocally the T3SS’s role in *Achromobacter* interactions with human macrophages and in infections in animal models.

The clinical isolates of *A. insuavis* and *A. xylosoxidans* investigated here induced macrophage cell death, but the extent of cytotoxicity was strain dependent. Of the three strains examined, cytotoxicity was rapid and extensive in *A. xylosoxidans* AC055, while *A. xylosoxidans* QV306 was the least toxic and *A. insuavis* AC047 displayed intermediate toxicity. These differences could merit further study, given that both *A. xylosoxidans* strains carry intact *sacUaxoU* operons encoding the AxoU phospholipase and its cognate chaperone, and the T3SS gene clusters are highly conserved in all three strains. The mechanism of cell death was attributed to pyroptosis, based on the hallmark cleavage of GSDMD and the release of IL-1β. T3SS effectors could contribute to modulation or evasion of pyroptosis, as this is a known phenomenon in other Gram-negative bacteria, like YopK in *Yersinia*.^51^ Bacteria can use other mechanisms for immune evasion,^52,53^ and it is possible that, rather than AC055 having a particularly unique virulence effector, QV306 may have a T3SS effector that enables delayed pyroptosis. Alternatively, the T3SS regulation may differ among the three strains, which could account for inter-strain variation in the degree of pyroptotic cell death.

The relatively low cytotoxicity of *A. xylosoxidans* QV306 made this strain useful to study the intracellular lifestyle of *Achromobacter*. After establishing that T3SS deletion did not affect internalization, we visualized internalized bacteria by immunostaining, demonstrating that they can survive for extended periods (at least up to 24 h) in a membrane-bound AcV. Experiments with specific membrane and fluid-phase fluorescent markers revealed that the AcV matures from an early phagosome into a late phagolysosome that is partially acidified, suggesting intracellular *Achromobacter* may interfere with the normal acidification process associated with the phagolysosome maturation pathway. Recapitulation of these experiments with the T3SS-defective *A. insuavis* AC047Δ*sctV* suggested that this secretion system may not be required for intracellular survival (see Limitations of the study below).

The *Achromobacter* strains induced pyroptosis by engaging either the NLRC4 or NLRP3 sensors, as demonstrated by infections in THP-1 cells with single and double knockouts in these sensors. However, our experiments could not distinguish whether effectors or structural T3SS components were detected by the inflammasome sensors. Detection of structural proteins of the assembled T3SS needle by NAIP/NLRC4-mediated sensing is a known phenomenon in *Salmonella*, *Shigella*, *Burkholderia*, and other bacteria.^40,54,55^ Because the *Achromobacter* isolates induced pyroptosis in NLRP3 KO THP-1 cells but not in the NLRC4/NLRP3 double KO cells, we conclude that the NLRC4 pathway is sufficient for pyroptosis induction, possibly by recognizing structural components of the T3SS that remain to be elucidated. Deletion of *fliC* decreased but did not abrogate cytotoxicity in NLRP3 KO THP-1, suggesting NLRC4 is not inducing pyroptosis solely through flagellin detection. The observation that NLRC4 was dispensable for T3SS-mediated pyroptosis in the presence of a functional NLRP3 was intriguing. It was recently demonstrated that in *Salmonella-*infected THP-1 cells, the bacterial flagellin activates NLRP3, while the PrgI(SctF) T3SS structural component activates NLRC4.^41^ The absence of flagellin did not affect the ability of AC047 to induce cytotoxicity in NLRC4 KO THP-1, ruling out this potential mechanism in our model. The lack of toxicity in the NLRC4/NLRP3 KO cell line infected with wildtype AC047 suggests that LPS detection by the non-canonical inflammasome^56^ is not involved in pyroptosis.

This study also demonstrated that AxoU can induce cell death independently of NLRC4 and NLRP3 and is not necessary for pyroptosis, since in its absence pyroptosis can still occur via either sensor. AxoU-induced cytotoxicity occurs even in the presence of a pan-caspase inhibitor, which abrogates cell death of macrophages infected with strains lacking AxoU. Moreover, *Bordetella bronchiseptica* (an evolutionary close relative of *Achromobacter)* can induce T3SS-dependent but caspase-1-independent necrosis.^57^ Induction of pyroptosis by AxoU via a pathway independent of NLRP3 or NLRC4 is unlikely. This is based on the observation that *A. xylosoxidans* AC055, which carries an intact *axoU* gene, failed to induce pyroptosis in the NLRC4/NLRP3 KO THP-1, suggesting that AxoU is probably causing necrosis. This conclusion is also consistent with the function of the ExoU, the AxoU homologue in *P. aeruginosa*, which causes ferroptosis,^58^ but is inhibitory of caspase-1 activity.^58,59^

T3SS-deficient *A. insuavis* was much less pathogenic than wildtype in systemic infection of *G. mellonella* larvae. Although inflammasome components are not completely conserved in insects, the cytosolic release of an inflammatory protein, the pro-phenoloxidase-containing crystal in *Drosophila*, which is crucial in the coagulation reaction for the entrapment and killing of microbes, is a caspase-dependent phenomenon that has been coined proto-pyroptosis.^60^ Since *G. mellonella* also produces phenoloxidase,^61^ it is possible that T3SS may act by enhancing proto-pyroptosis, resulting in the upregulated release of this enzyme, causing the death of the larvae. However, in a mammalian host, we show that proinflammatory lung tissue damage occurred in mice infected with wildtype but not in those infected with the T3SS-deficient mutant. These findings agree with recent studies showing that *A. xylosoxidans* induced acute lung inflammation in mice,^47,62^ which is consistent with the idea that macrophages undergo pro-inflammatory cell death by pyroptosis. Using an intratracheal mouse infection model, Wills et al.^47^ have shown that a defective T3SS in *A. xylosoxidans* limits blood dissemination of the infection, which may also contribute to reduce systemic inflammation and facilitating the resolution of the infection.

In summary, this study shows that *Achromobacter* species can interact with human macrophages to induce pyroptosis by engaging NLRC4 or NLRP3 sensors in a T3SS-dependent manner. We also show that pyroptosis is AxoU-independent and does not require the RTX adhesin, both of which are only present in *A. xylosoxidans* isolates,^21,22^ demonstrating that the T3SS is a common denominator of pathogenicity in *Achromobacter* species. We propose that *Achromobacter* interactions with innate immune cells are particularly critical to determine infection and inflammation under conditions where the host is partially immunosuppressed, such as in individuals with cystic fibrosis or other immunocompromising conditions, where *Achromobacter* species find a favorable niche to colonize and infect.

### Limitations of the study

One caveat to be considered in our study is the difficulty to kill extracellular bacteria during the infection assays, which hampers the analysis of bacterial intracellular survival in the AC047Δ*sctV* mutant to unequivocally assess if the T3SS is involved. Others have used antibiotics at high concentrations^22^; however, in our opinion, these approaches are risky since it is difficult to avoid the risk that the antibiotics can enter the host eukaryotic cell and kill intracellular bacteria. In previous work on *Burkholderia cenocepacia*, we have overcome this limitation by constructing a deletion mutant in an efflux pump gene cluster responsible for aminoglycoside resistance.^63^ Experiments are underway in our laboratory to assess if a similar approach may be used for *Achromobacter* isolates. Another limitation of this study is the mechanistic characterization of the role of AxoU in macrophage toxicity and elicitation of inflammation. Our data support the notion that AxoU is not involved in pyroptosis, but its mechanism of cytotoxicity and a potential role in inflammation by pyroptosis-independent mechanisms were not elucidated. We used here intranasal mouse infections to assess the role of the T3SS in promoting lung infection and inflammation. Although our experiments have been informative establishing the role of T3SS in the pathogenicity of AC047, the model does not mimic the infection in humans, especially in individuals with cystic fibrosis where *Achromobacter* sp. establish chronic lung infections. Moreover, genetic KO mice in the various components of the inflammasome pathways would add more mechanistic understanding to the function of the T3SS during infection.

## Star★Methods

Detailed methods are provided in the online version of this paper and include the following:

- Key resources table
- Resource availability
  ○ Lead contact
  ○ Materials availability
  ○ Data and code availability
- Experimental Model and Subject Participant Details
  ○ Cell lines
  ○ Mice
  ○ Galleria mellonella
  ○ Bacterial strains
- Method Details
  ○ Mutagenesis
  ○ *In vitro* Infection
  ○ LDH assay
  ○ Propidium iodide uptake assay
  ○ Immunoblotting
  ○ ELISAs
  ○ Immunostaining
  ○ Live imaging of infected cells
  ○ Cblaster
- Quantification and Statistical Analysis

## Star★Methods

**Table:** Key Resources Table

REAGENT or RESOURCE	SOURCE	IDENTIFIER
Antibodies		
Gasdermin-D; working dilution 1:1000	Cell Signaling Technology	Cat #93709; RRID:AB_2800210
Cleaved Gasdermin-D (N terminus); working dilution 1:1000	Cell Signaling Technology	Cat# 36425; RRID:AB_2799099
GAPDH; working dilution 1:2000	Abcam	Cat# ab8245; RRID:AB_2107448
Caspase-1 p20; working dilution 1:1000	Cell Signaling Technology	Cat# 4199; RRID:AB_1903916
EEA1; working dilution 1:1142	Invitrogen	Cat# PA1-063A; RRID AB_2096819
LAMP-1; working dilution 1:800	Abcam	Cat# ab24170; RRID:AB_775978
LCB3; working dilution 0.5 μg/mL	Invitrogen	Cat# L10382
IgG control; Equivalent in μg/ml to LAMP-1 ab	Abcam	Cat# Ab171870; RRID:AB_2687657
Rabbit IgG; working dilution 1:10000	LI-COR Biosciences	Cat# 925-32211, RRID:AB_2651127
Mouse IgG; working dilution 1:10000	LI-COR Biosciences	Cat# 926-32210; RRID:AB_621842
Rabbit IgG (Alexa 488); working dilution 1:2000	Abcam	Cat# ab150077; RRID:AB_2630356
Bacterial and virus strains		
*Achromobacter xylosoxidans*	UK Health Security Agency;^64^	AC011
*Achromobacter* sp.	UK Health Security Agency;^64^	AC035
*Achromobacter insuavis*	UK Health Security Agency;^64^	AC047
*Achromobacter xylosoxidans*	UK Health Security Agency;^64^	AC055
*Achromobacter xylosoxidans*	UK Health Security Agency;^64^	AC088
*Achromobacter xylosoxidans*	American Type Culture Collection;^64^	ATCC27061
*Achromobacter xylosoxidans*	Lab collection	QV306
*Achromobacter insuavis* AC047*ΔsctV*	This work	AC047Δ*sctV*
*Achromobacter insuavis* AC047Δ*axlG*	This work	AC047Δ*axlG*
*Achromobacter insuavis* AC047Δ*sctX*	This work	AC047Δ*sctX*
*Achromobacter insuavis* AC047Δ*fliC*	This work	AC047Δ*fliC*
*Achromobacter insuavis* AC047Δ*hcp*	This work	AC047Δ*hcp*
*Achromobacter xylosoxidans* AC055:pGPI-SceI*-axoU*	This work	55:pGPI-*axoU*
*Achromobacter xylosoxidans* AC055:pGPI-SceI*-sctV*	This work	55:pGPI-*sctV*
*Achromobacter xylosoxidans* QV306:pGPI-SceI*-sctV*	This work	QV:pGPI-*sctV*
*Burkholderia cenocepacia* Δ*atsR*	Aubert et al.^65^	DFA35
*Escherichia coli* F - φ80*lacZ* M15 *endA1 recA1 supE44 hsdR17(r_K_^-^ m_K_^+^)* *deoR thi-1 nupG supE44 gyrA96 relA1* Δ(*lacZYA-argF*)U169, *λ*-	Laboratory stock	DH5*α*
*Escherichia coli Δasd thi-1 thr-1 leuB26 tonA21 lacY1 supE44 recA; integrated RP4-2 Tcr::Mu ΔaphA (λpir^+^)*	López et al.^66^	RHO3
*Escherichia coli F-, araD, Δ(lac pro), argE(Am), recA56, Rif^R^, gyrA λpir*	Miller et al.^67^	SY327*λpir*
Biological samples		
Human buffy coats for isolation of human primary monocytic macrophages	Northern Ireland Blood Transfusion Service	Project Reference number 2019/09
Chemicals, peptides, and recombinant proteins		
RPMI 1640 Medium	ThermoFisher Scientific	Cat# 11875093
Fetal Bovine Serum, qualified, heat inactivated, Brazil	ThermoFisher Scientific	Cat# 10500064
Penicillin-Streptomycin (10,000 U/mL)	ThermoFisher Scientific	Cat# 15140122
IMDM medium	ThermoFisher Scientific	Cat# 12440053
Phorbol 12-myristate 13-acetate	Sigma-Aldrich	Cat# P8139-1MG
Recombinant Human GM-CSF	Peprotech	Cat# 300-03
Agar	Melford	Cat# A20250
Ammonium chloride	Biosciences	Cat# RC-015
Ammonium sulfate	Honeywell	Cat# A5132-1KG
Bafilomycin A1	Merck	Cat#SML1661
Calcein blue	ThermoFisher Scientific	Cat#C1429
CD14 Microbeads 2ML	Miltenyi Biotec	Cat# 130-050-201
Chloramphenicol	Sigma-Aldrich	Cat# C0378-25G
Cytochalasin D	Sigma-Aldrich	Cat#C2618-200UL
Dextran	ThermoFisher Scientific	Cat# D1821
DMSO	Sigma-Aldrich	Cat# D8418
Fluoroshield with 1,4-Diazabicyclo[2.2.2]octane	Sigma-Aldrich	Cat# F6937-20ML
Glycerol	Sigma-Aldrich	Cat#G5516
High salt LB	Melford	Cat# L24040
Lysotracker	Invitrogen	Cat# L7526
Paraformaldehyde Solution, 4% in PBS	ThermoFisher Scientific	Cat# 15670799
Phosphate buffered saline	Sigma-Aldrich	Cat# P4417-100TAB
Potassium phosphate monobasic	Sigma-Aldrich	Cat# P5379-500G
Potassium nitrate	Sigma-Aldrich	Cat# P8291-KG
Propidium iodide	Sigma-Aldrich	Cat# P4864-10ML
Sodium dihydrogen orthophosphate dihydrate	GPR	Cat#301324Q
Tetracycline hydrochloride	Sigma-Aldrich	Cat#T3383-100G
Triton X-100	Bio-Rad	Cat# 1610407
Tryptone	Sigma-Aldrich	Cat# T9410-1KG
Tween 20	Sigma-Aldrich	Cat# P1379-500mL
VX-765	MedChemExpress	Cat# HY-13205
Z-VAD-FMK	InvivoGen	Cat# tlrl-vad
Critical commercial assays		
ROCHE Cytotoxicity Detection Kit (LDH)	Scientific Laboratory Supplies	Cat# 11644793001D2
Human IL-1 beta/IL-1F2 DuoSet ELISA	R&D Systems	Cat# DY201-05
Mouse CXCL1/KC DuoSet ELISA	R&D Systems	Cat# DY453-05
Mouse IL-6 DuoSet ELISA	R&D Systems	Cat# DY406-05
DuoSet ELISA Ancillary Reagent Kit 2	R&D Systems	Cat# DY008B
Q5® Hot Start High-Fidelity DNA Polymerase	New England BioLabs	Cat# M0493S
Trans-Blot Turbo RTA Mini 0.2 μm Nitrocellulose Transfer Kit, for 40 blots	Bio-Rad	Cat# 1704270
Experimental models: Cell lines		
THP-1	American Type Culture Collection	Cat# TIB-202™
NLRP3 KO THP-1	Schmid-Burgk et al.^42^	N/A
NLRC4 KO THP-1	Gram et al.^41^	N/A
NLRC4/NLRP3 KO THP-1	Gram et al.^41^	N/A
Experimental models: Organisms/strains		
Mice	Charles River Laboratories, UK	C57BL/6
*Galleria mellonella* larvae	UK Waxworms Ltd.	N/A
Oligonucleotides		
5’-GAACTGGATTCCCGACCTGTT; for Sanger sequencing	Eurofins Scientific	*Achromobacter* sp. *nrdA* forward
5’-TTCGATTTGACGTACAAGTTCTGG, for Sanger sequencing	Eurofins Scientific	*Achromobacter* sp. *nrdA* reverse
5’-ATGCGCCAGTTCGACTACATCTCCGAAATGATG, for Sanger sequencing	Eurofins Scientific	*A. xylosoxidans sctN* forward
5’-GCCGCCCCCACGATGGCG, for Sanger sequencing	Eurofins Scientific	*A. xylosoxidans sctN* reverse
Additional oligonucleotides are listed in Table S1		
Recombinant DNA		
Suicide plasmid vector, R6Kγ origin of replication, Mob^+^, carries a I-*Sce*I endonuclease site; Cm^R^	Flannagan et al.^50^	pGPI-SceI-XCm
*sctV*(*A. xylosoxidans)* mutagenic plasmid	This study	pGPI-SceI-XCm-*sctV*(Ax)
*sctV*(*A insuavis)* mutagenic plasmid	This study	pGPI-SceI-XCm-*sctV*(Ai)
*sctX* mutagenic plasmid	This study	pGPI-SceI-XCm-*sctX*
*axlG* mutagenic plasmid	This study	pGPI-SceI-XCm-*axlG*
*hcp* mutagenic plasmid	This study	pGPI-SceI-XCm-*hcp*
*axoU* mutagenic plasmid	This study	pGPI-SceI-XCm-*axoU*
Expresses the I-*Sce*I endonuclease, *sacB* gene; Tet^R^	Flannagan et al.^50^	pDAI-SceI-sacB
Expression vector (untagged); Tet^R^	Aubert et al.^68^	pDA12
pDA12 carrying *sctX*	This study	pDA12-*sctX*
Expression vector to construct C-terminal FLAG-tagged protein fusions; Tet^R^	Aubert et al.^68^	pDA17
pDA17 carrying *axlG*_FLAG_	This study	pDA17-*axlG*
pDA12 encoding the mCherry red fluorescent protein; Tet^R^	J. Torres Bustos	pJT04
*ori*_pBBR_ *mob^+^,* Cm^R^, dsRed gene	Vergunst et al.^69^	pIN62
pIN62, dsRed gene replaced by the mCherry red fluorescent protein gene; Cm^R^	J. Torres Bustos	pJT05
Software and algorithms		
Prism v. 9.4	GraphPad Software, LLC	RRID:SCR_002798
Benchling Software	https://www.benchling.com/	RRID:SCR_013955
cblaster v1.3.16	Gilchrist et al.^32^	N/A
Other		
Transwells	Sarstedt	Cat# 83.3932.040
LS Columns 25/PK	Miltenyi Biotec	Cat# 130-042-401
Ficoll® Paque Plus	Sigma-Aldrich	Cat# 17-1440-03
PolarStar plate reader	BMG Labtech	Cat# 415-201
Trans-Blot Turbo Transfer Starter System	Bio-Rad	Cat# 17001918
SP8 and Stellaris confocal microscopes	Leica	N/A

## Resource Availability

### Lead contact

Further information and requests for resources and reagents should be directed to and will be fulfilled by the lead contact, Miguel A. Valvano (m.valvano@qub.ac.uk).

### Materials availability

All unique reagents generated in this study are available from the lead contact without restriction.

## Experimental Model and Subject Participant Details

### Cell lines

Wildtype THP-1 cells, and THP-1 with CRISPR-based knockouts of NLRP3,^42^ NLRC4, and NLRC4/NLRP3^41^ were each cultured in RPMI 1640 supplemented with 10% heat-inactivated FBS and 1% penicillin-streptomycin. All knockout cell lines have been previously validated.^41,42^ Cells were seeded at a density of 2 x 10^5^ cells/ml and differentiated in 80 ng/mL PMA for 3 days, then rested overnight without PMA before infection. Human monocyte-derived macrophages (HMDMs) were obtained from buffy coats provided by the Northern Ireland Blood Transfusion Service (Project Reference number 2019/09) according to ethical approval by the Ethics Committee of the Faculty of Medicine, Health and Life Sciences, Queen’s University Belfast (Reference MLHS 19_22). Buffy coats were separated by Ficoll-Paque gradient density fractionation, and monocytes positively selected by incubation with CD14^+^ beads. Isolated cells were cultured in IMDM 10% FBS and 1% penicillin-streptomycin 50 ng/mL GM-CSF for seven days. Cells were plated at a density of 5 x 10^5^ cells/ml with GM-CSF and infected the next day. For all cell lines used, cells were refreshed with antibiotic-free media 1 h prior to infection.

### Mice

C57BL/6 mice, originally purchased from Charles River Laboratories (UK), were bred in house. Animal work was conducted according to the Animals Scientific Procedures Act (1986). The research was ethically reviewed by both the University Animal Welfare and Ethical Review Body (AWERB) and the Northern Ireland Dept of Health. The research was carried out under approved project licenses PPL2807. Adult female C57BL/6 mice were used in these studies. Mice were given free access to food and water and subjected to 12-h light/dark cycle. A log phase culture of *A. insuavis* AC047 and AC047Δ*sctV* was washed and resuspended in sterile endotoxin-free PBS and diluted based on 1 OD6_00nm_ is equal to 1x10^10^ CFU/ml. Prepared inoculums were plated on MTG plates to confirm CFU dose delivered. Groups of n = 5 mice were briefly anesthetized with a mixture of xylazine hydrochloride and ketamine injected intraperitoneally, and inoculated intranasally with either with 10^7^,10^8^or 10^9^ CFU of AC047 or 10^8^ AC047Δ*sctV*or PBS (in 20 μL). Mice were sacrificed at 24 h p.i., unless a predefined humane endpoint was reached prior to that. Lungs were collected into 2 mL of PBS and homogenates were serially diluted and plated on MTG agar overnight at 37°C for quantification of CFUs. Homogenates were also analyzed for IL-6 and KC by ELISA (R&D Systems). To directly compare the wild type and mutant, nine-plus-week old female C57BL/6 mice were infected intranasally with 10^7^ of AC047 or AC047Δ*sctV* (n = 10 per group). Mice were sacrificed 24 h post-infection and n = 7 were analyzed for bacterial load and IL-6 and KC using ELISA (R&D systems). A further n = 3 animals were analyzed using histology. The lungs were inflated with paraformaldehyde (PFA), fixed in PFA for 24 h and dehydrated. The specimens were then embedded in paraffin, cut in 5 μm sections, and stained with hematoxylin-eosin. The protocols for histology scoring have been described before, for alveolar wall damage (mean linear intercept)^70^ and septa thickness^71^ and here they are an average of 5 fields of view of 2 sections for each condition at magnification ×20.

### Galleria mellonella

*Galleria mellonella* larvae (UK Waxworms Ltd.) were stored at 16°C and used within 3 weeks of delivery. Prior to infection, larvae were weighed to ensure they were between 0.25 and 0.3 g and visually inspected to ensure they were healthy and had no signs of melanization indicating prior infection or stress. Infection was carried out using a Hamilton syringe which was sterilized using 70% ethanol. A hypodermic needle (BD microlance, 30-gauge, 13 mm) which was changed between each experimental condition was fitted and 10 μL of the respective bacterial suspension was aspirated and injected into the rear proleg of each larva. Ten larvae were infected for each isolate and the infections were repeated three times; in addition, a group was injected with the same volume of PBS to control for inoculation injury. We did dose-response curves to determine the optimal CFU for injection. Larvae were injected as described,^72^ with 10 μL sterile PBS or 10 μL bacteria of total CFU 10^6^ of wildtype AC047 or AC047Δ*sctV*. Larvae were placed at 37°C and assessed daily for indications of morbidity or mortality.

### Bacterial strains

Clinical isolates used in this work were part of a collection of *Achromobacter* species sputum isolates originated from individuals with cystic fibrosis, which were obtained from the Antimicrobial Resistance and Healthcare Associated Infections Reference Unit of the UK Health Security Agency. Strains were grown in a semi-defined mineral tryptone glycerol (MTG) medium, as previously described.^64^ We used *nrdA* sequencing to confirm the species assignments of each strain used. For immunostaining analyses, we conjugated pJT04, a vector expressing mCherry with *tetAR* cassette, into AC047Δ*sctV*. Additionally, QV306 was conjugated with pJT05, a vector expressing mCherry, for trafficking analysis. All strains used in this work are listed in the key resources table.

## Method Details

### Mutagenesis

A protocol adapted from *Burkholderia*^50^ was used to generate clean deletions in AC047 and disruption mutants in our *A. xylosoxidans* strains. Briefly, we created suicide vectors containing upstream and downstream regions from our target genes. The vectors had a Sce-I excision site, so that after integration we could introduce an excision vector encoding the restriction enzyme to disrupt the gene. We checked putative mutants by PCR with primers upstream of the US and downstream of the DS, and amplicons of the correct size were sequenced. Suicide and excision vectors were sequentially introduced into *Achromobacter* via conjugation with RHO3, an auxotrophic donor strain requiring diaminopimelic acid.^66^ The plasmids used for each mutant are listed in key resources table. We used plasmids derived from *Bordetella* (pDA17 encoding a C-terminal FLAG tag and pDA12 for untagged) for complement expression.^73^ Constructs were generated by Gibson assembly,^74^ with a few created by restriction/ligation (see key resources table).

### *In vitro* Infection

*Achromobacter* were prepared by 3 washes in RPMI and adjusted by OD to MOIs of 20 and 80, as indicated. *Burkholderia* was adjusted to an MOI of 30. Heat-killed bacteria (60°C 20 min) served as controls. Cells were infected with bacteria and spun down at 200 xg for 5 min to synchronize infection. Cells were incubated at 37°C at various times from 15 min up to 5 h p.i. For transwell experiments, HMDMs were plated in 24-well plates and bacteria were added in the upper chamber in 20% total media volume at the same MOI as during direct infection, following the protocol of Flaherty and Lee.^34^

### LDH assay

Supernatants from infected cells were evaluated using the Roche LDH assay kit. This assay detects lactate dehydrogenase in the medium, which indicates cell membrane rupture. The assay reagents were prepared, and 50 μL were added to 50 μL of each sample media. Data represent four technical replicates per biological replicate, with uninfected and 1% Triton X-100-treated (15 min) cells serving as the low (0%) and high (100%) controls respectively. For caspase inhibition, cells were treated with 10 μg/mLZ-VAD-FMK (or equivalent volume DMSO) for 1 h pre-infection, and the same concentration during a 3-h infection.

### Propidium iodide uptake assay

HMDM were plated at a density of 5 x 10^4^ cells per 100ul in transparent-base, black-sided 96 well plates. On the day of infection, 2 ng/mL propidium iodide was added to all test condition wells along with the bacteria. Propidium iodide-free cells were used as a blank, and 1% Triton X-100 cells provided a fluorescence normalization reading. We took fluorescence readings every 20 min using a PolarStar plate reader with 5% CO2 at 37°C. For the internalization and inhibition assays, cells were treated with 5 μg/mL cytochalasin D or 40 μM VX-765 (or equivalent volume DMSO) for 1 h pre-infection, and the same concentration during infection.

### Immunoblotting

Lysates were prepared by washing cells with PBS twice, then adding 2% SDS 66mM Tris lysis buffer (10 μL per 100,000 cells) and scraping lysates into Eppendorf tubes on ice. Protein concentration was determined by BCA, and equivalent lysates loaded onto SDS-PAGE gels. We used a Trans-Blot Turbo Transfer then blocked with casein-TBS for 1 h. Blots were incubated in anti-GSDMD primary antibody in TBS-T, rocking overnight at 4°C, washed thrice with TBS-T, then incubated at room temperature in secondary antibody conjugated to fluorophore for 1 h. Blots were visualized using an LI-COR system. Blots were stripped and re-probed with anti-GAPDH, a loading control. Antibodies, codes, and concentrations are listed in the key resources table.

### ELISAs

HMDMs were infected as described above. At 5-h post-infection, plates were spun down (5 min 200 xg), and supernatants were collected and immediately used for analysis. We used the R&D Technologies IL-1β ELISA kit (key resources table), following manufacturer instructions and including 4 technical replicates per biological replicate. The mouse cytokine levels were detected in the same way, using kits for IL-6 and KC. Details follow about lung homogenate preparation.

### Immunostaining

UV-sterilized coverslips were placed in 12-well cell culture plates prior to seeding of THP-1 and HMDM. Infections were carried out as described above, and at various time points the coverslips with cells were retrieved, fixed in 4% paraformaldehyde, washed with PBS, and then placed in 14mMammonium chloride overnight at 4°C to quench free aldehyde groups. Cells were permeabilized using 0.5% saponin,^75^ and then incubated in a humidified chamber at room temperature for 1 h with primary antibodies (key resources table). Coverslips were washed twice in PBS, then incubated 1 h in secondary goat anti-rabbit Alexa Fluor 488 antibody, as above. Slides were imaged using SP8 confocal and Stellaris-5 confocal microscopy, images taken at random.

### Live imaging of infected cells

THP-1s were seeded directly into 8-well chamber slides (Ibidi GmbH). Infections were carried out as described above, at various timepoints the cells were retrieved and incubated with fluid phase markers (key resources table). Cells were immediately imaged by Stellaris-5 confocal microscopy.

### Cblaster

The synteny of *axlG* and *sctA* genes was determined using cblaster v1.3.16.^32^ Briefly, we determined the co-occurrence ofthe genes using an NCBI’s non-redundant protein sequences (nr) database, with the search term “Achromobacter” accessed on 10.12.2022.

## Quantification and Statistical Analysis

Unless specifically noted, all data are representative of 3 separate biological repeats. Experimental group assignment was determined by random designation. Statistical analyses were performed using GraphPad Prism software v9.4. Error bars represent ±standard deviation (S.D.) calculated using Prism. Specific statistical tests used were paired Student’s t-tests, two-way ANOVA, and Mantel-Cox Log Rank, as specified in the figure legends. p values <0.05 were considered statistically significant. Investigators were not blinded to group assignment during experimental procedures or analysis.

## Supplementary Material

Supplemental information can be found online at https://doi.org/10.1016/j.celrep.2023.113012.

Supplemental information

## Data Availability

The data reported in this paper will be shared by the lead contact upon request. This paper does not report original code. Any additional information required to reanalyze the data reported in this paper is available from the lead contact upon request.

## In brief

Turton et al. show that, upon phagocytosis, the *Achromobacter* T3SS activates either NLRC4 or NLRP3 sensors, causing pro-inflammatory cell death in human macrophages, and mediates lung damage in infected mice, together demonstrating that *Achromobacter* bacteria overcome phagocytosis via pro-inflammatory cell death induction by redundant T3SS-dependent mechanisms.

## Highlights

- The *Achromobacter* T3SS induces pyroptosis in human macrophages
- The inflammasome sensors NLRC4 or NLRP3 mediate *Achromobacter-induced* pyroptosis
- *Achromobacter* bacteria traffic intracellularly to a late phagolysosome compartment
- *Achromobacter* T3SS is required to damage lung structure in a mouse infection model
