# Supplementary material for: The Achromobacter type 3 secretion system drives pyroptosis and immunopathology via independent activation of NLRC4 and NLRP3 inflammasomes
Source: Cell Rep. Author manuscript; Available in PMC 2023 Aug 25. (PMC7614980; doi:10.1016/j.celrep.2023.113012)
Supplement: Supplemental information [file EMS185177-supplement-Supplemental_information.pdf]

Cell Reports, Volume *42*

Supplemental information

The *Achromobacter* type 3 secretion system drives pyroptosis and immunopathology via independent activation of NLRC4 and NLRP3 inflammasomes

Keren Turton, Hannah J. Parks, Paulina Zarodkiewicz, Mohamad A. Hamad, Rachel Dwane, Georgiana Parau, Rebecca J. Ingram, Rebecca C. Coll, Clare E. Bryant, and Miguel A. Valvano

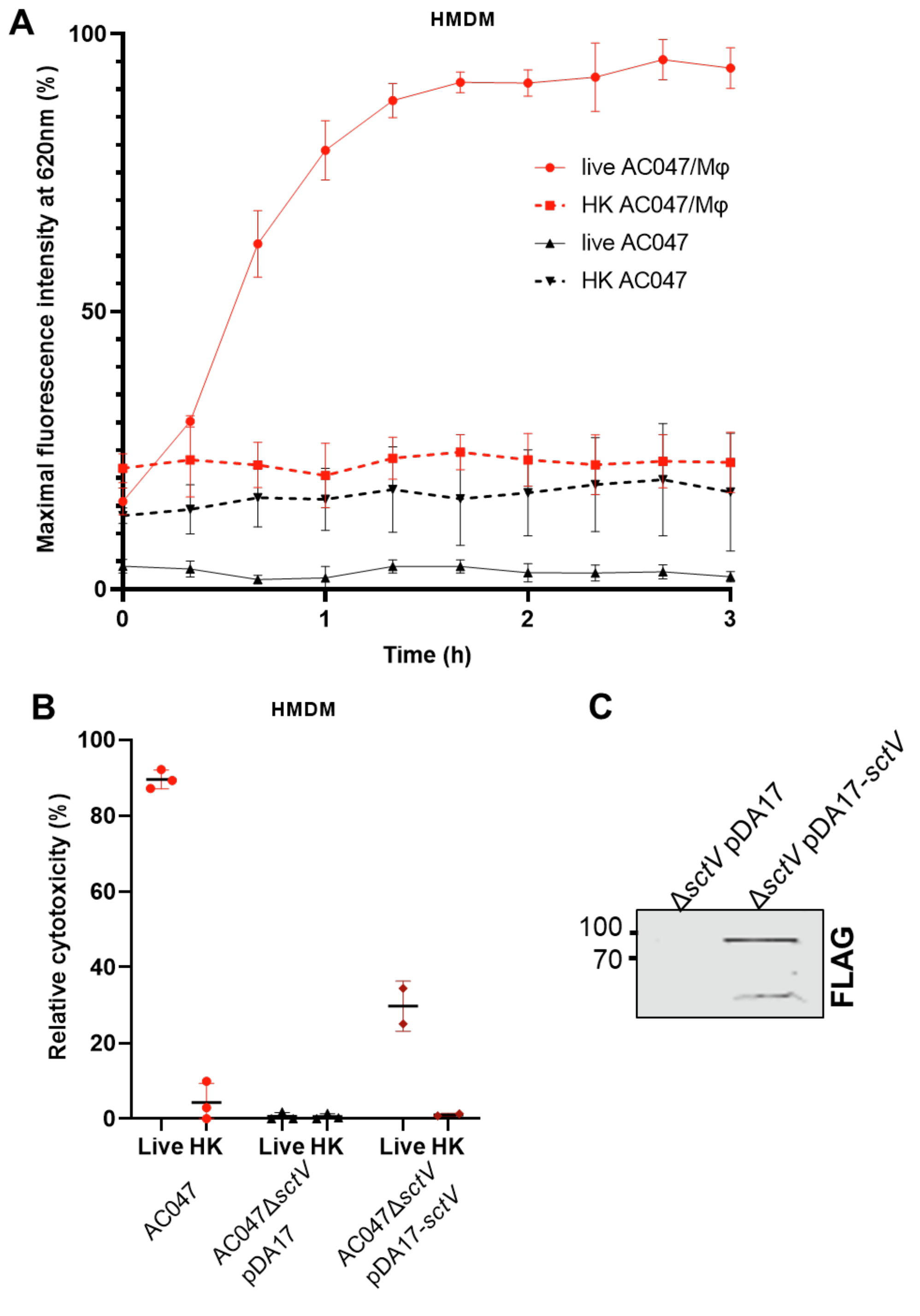

Figure S1. Propidium iodide uptake assay controls. Infections were carried out with MOI = 20 and followed for 3 h. **A.** PI assay of live and HK AC047 in the presence or absence of HMDM (Mφ). **B.** LDH assay and immunoblot (panel **C**) of FLAG to assess complementation with pDA17-sctV-FLAG. Data in panels A and B are represented as mean ± SD from at least three biological replicates with four technical replicas each.

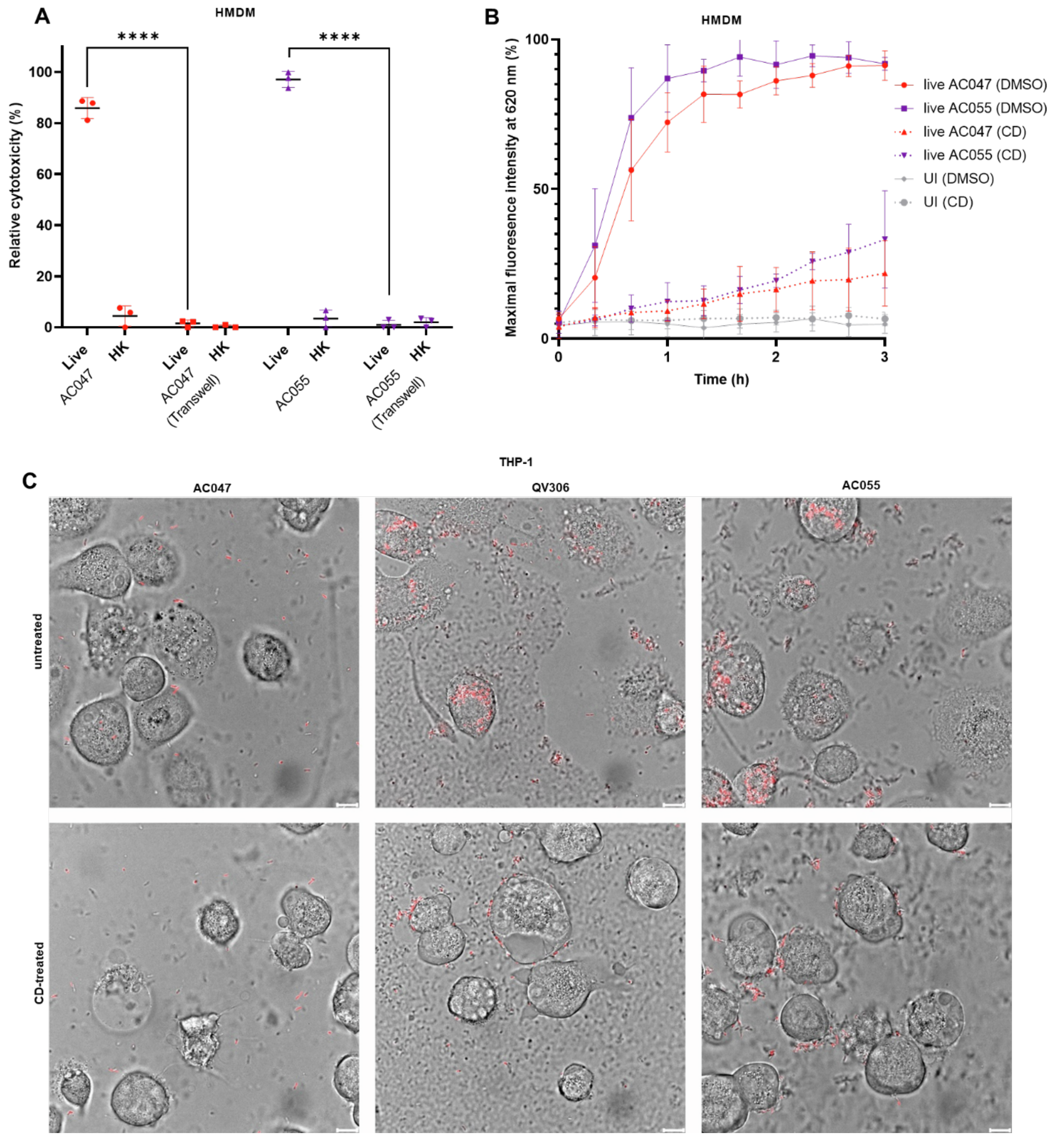

Figure S2. Macrophage cytotoxicity is contact-dependent and enhanced by internalization. **A**. LDH release of HMDMs infected with live and heat-killed (HK) AC047 and AC055, comparing direct infection and infection using transwells at 5 h p.i., MOI = 20. Data are represented as mean ± SD from at least three biological replicates. ****, $p < 0.0001$ by t-test. **B.** Propidium iodide uptake assay of HMDMs, untreated or treated with 5 µg/ml cytochalasin D. Data are represented as mean ± SD from four technical replicates for each of three biological replicates. **C.** THP-1 macrophages 5 h p.i. with mCherry labelled QV306, AC047 and AC055, untreated or treated with 5 µg/ml cytochalasin D. Images taken with × 100 magnification on Leica Stellaris-5 confocal microscope. MOI = 80 (QV306) and 20 (AC047, AC055). Scale bar = 10 µm.

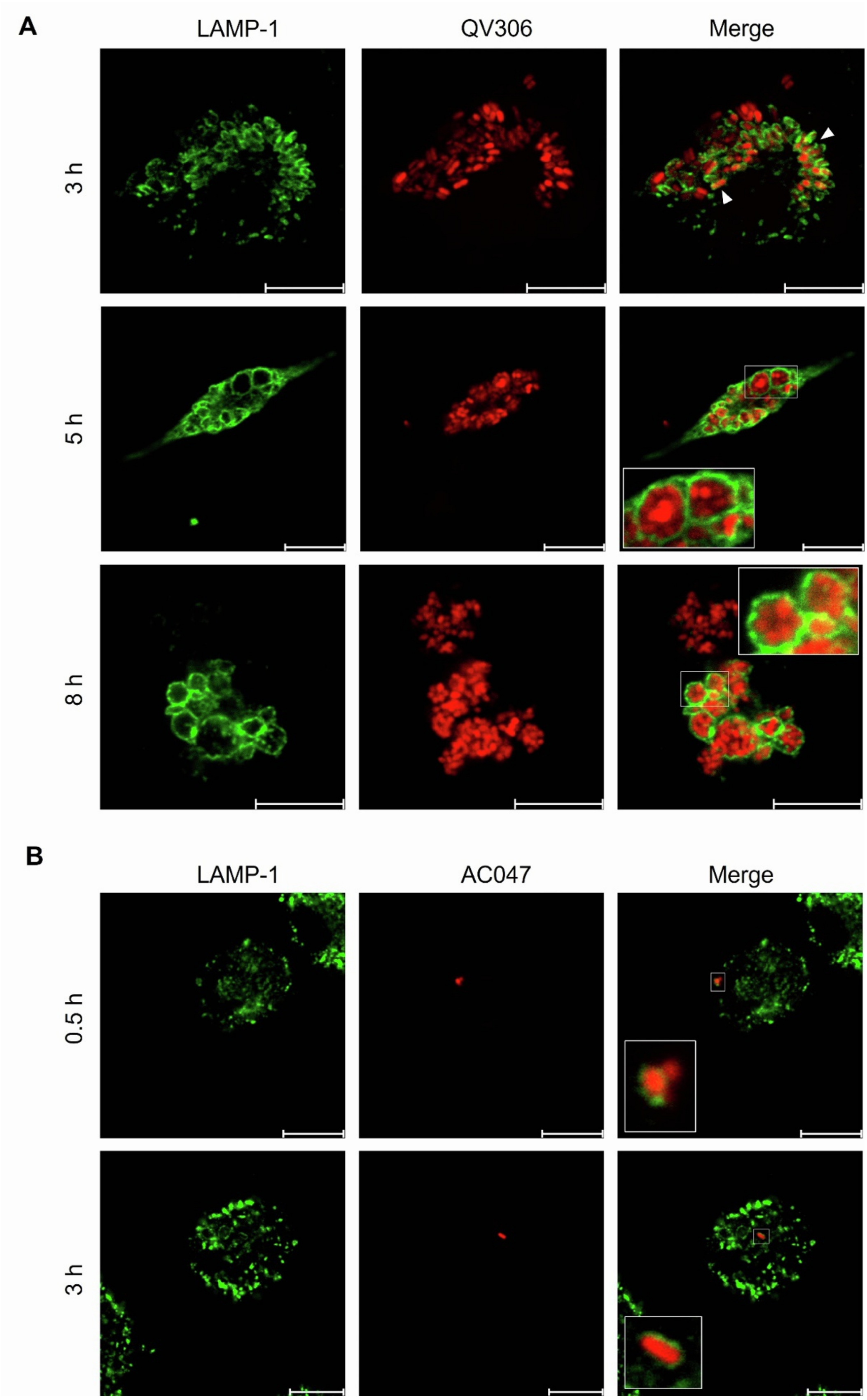

Figure S3. The *Achromobacter*-containing vacuole (AcV) colocalizes with LAMP-1 in HMDMs and in THP-1 macrophages infected with QV506 and AC047, respectively. A. THP-1 macrophages infected with live *A. xylosoxidans* QV306 at 3-, 5- and 8-h p.i. contain bacteria in AcVs that colocalize with LAMP-1. Images taken with × 63 magnification on Leica SP8 confocal microscope. MOI = 80. **B.** THP-1 macrophages infected with live *A. insuavis* AC047 at 0.5- and 3-h p.i. contain AcVs that colocalize with LAMP-1. MOI = 20. Images taken with × 100 magnification on Stellaris-5 confocal microscope. Scale bar = 10 µm.

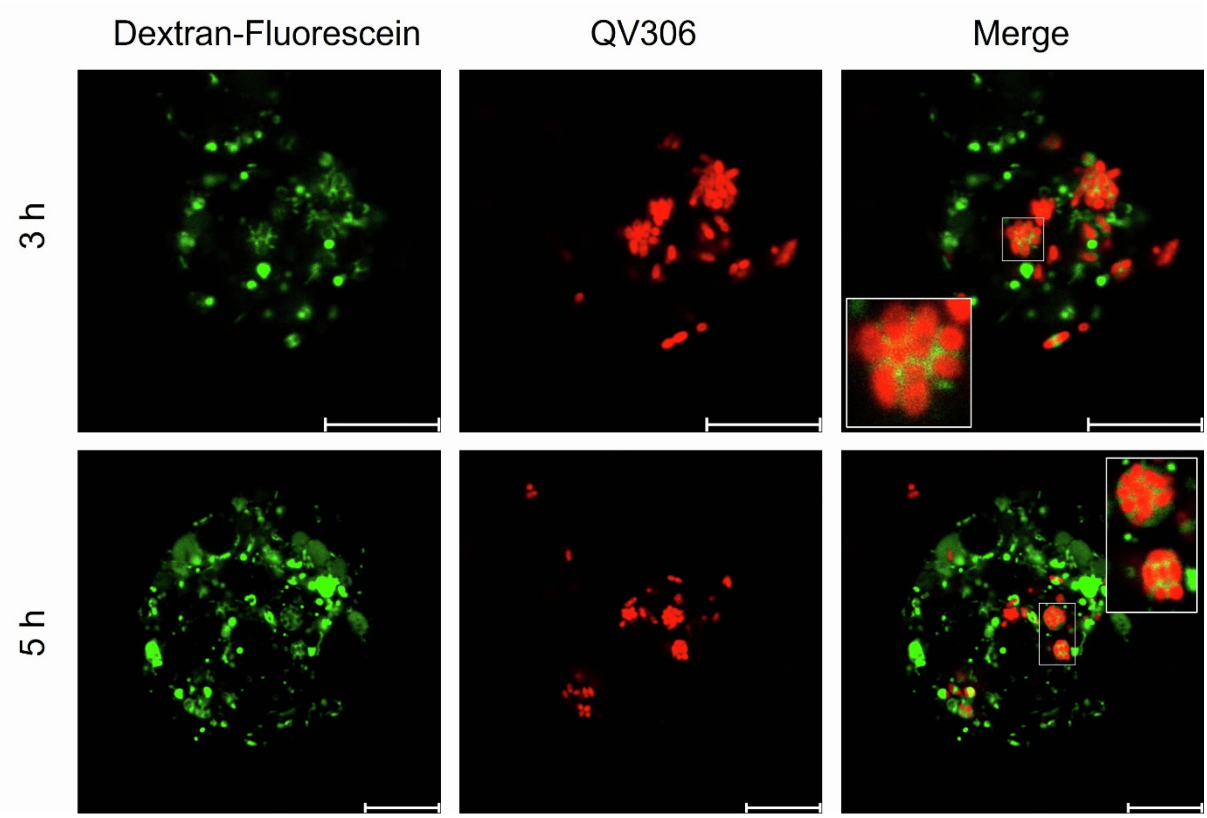

Figure S4. The QV306 AcV co-localizes with Dextran-fluorescein. THP-1 macrophages 3 and 5 h p.i. were pre-treated before infection with the fluid phase marker dextran fluorescein. Images taken with × 100 magnification on Leica Stellaris-5 confocal microscope. MOI = 80. Scale bar = 10 µm.

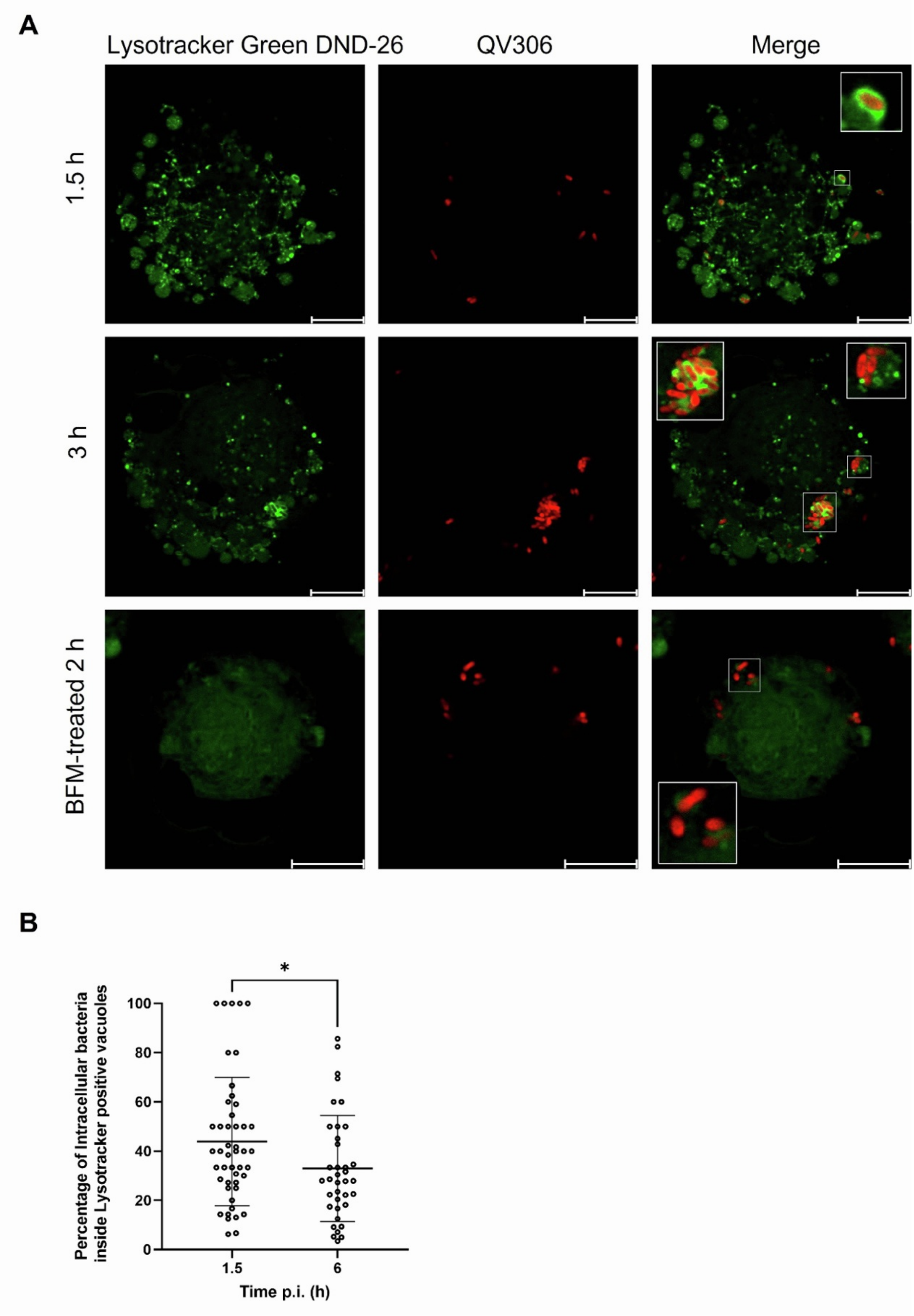

Figure S5. The QV306 AcV colocalize in acidic and non-acidic compartments. A. THP-1 macrophages 1.5- and 3-h p.i. pretreated with the fluid phase marker Lysotracker green DND. In some experiments, acidification was inhibited using bafilomycin (BFM) at 2 h p.i. Cells were immediately imaged on the Leica Stellaris-5 confocal microscope, × 100 magnification. MOI = 80. Scale bar = 10 µm. **B**. The percentage of intracellular bacteria present in lysotracker positive compartments was assessed in 80 macrophages. Data are represented as mean ± SD. *, $p < 0.05$ by Mann-Whitney test. Error bars represent standard deviation from the mean; datasets from two biological replicates.

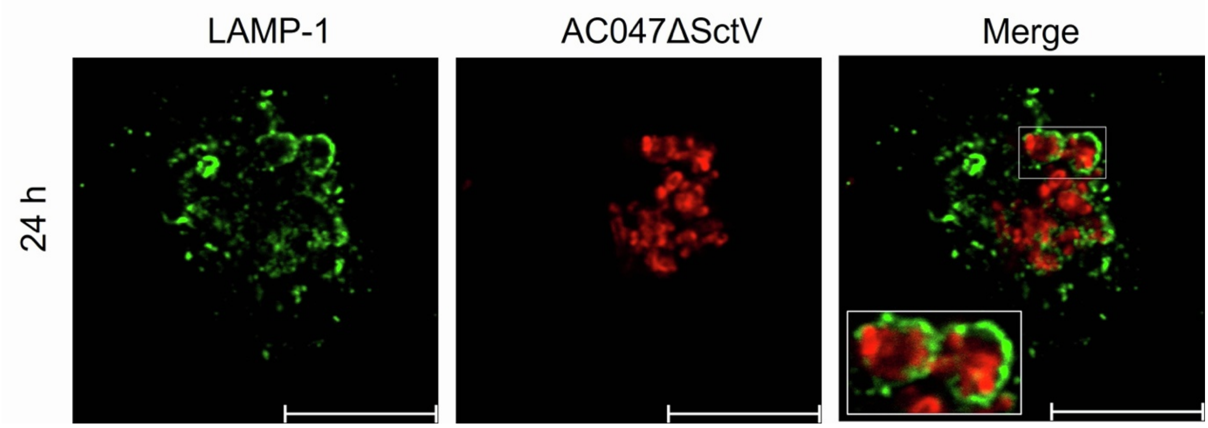

Figure S6. mCherry-labelled AC047ΔSctV 24 h post-infection of HMDMs. HMDM macrophages infected with live *A. insuavis* AC047 Δ*sctV* mutant at 24-h p.i. contain AcVs that colocalise with LAMP-1. (MOI = 20). Images taken with × 100 magnification on Stellaris-5 confocal microscope. Scale bar = 10 µm.

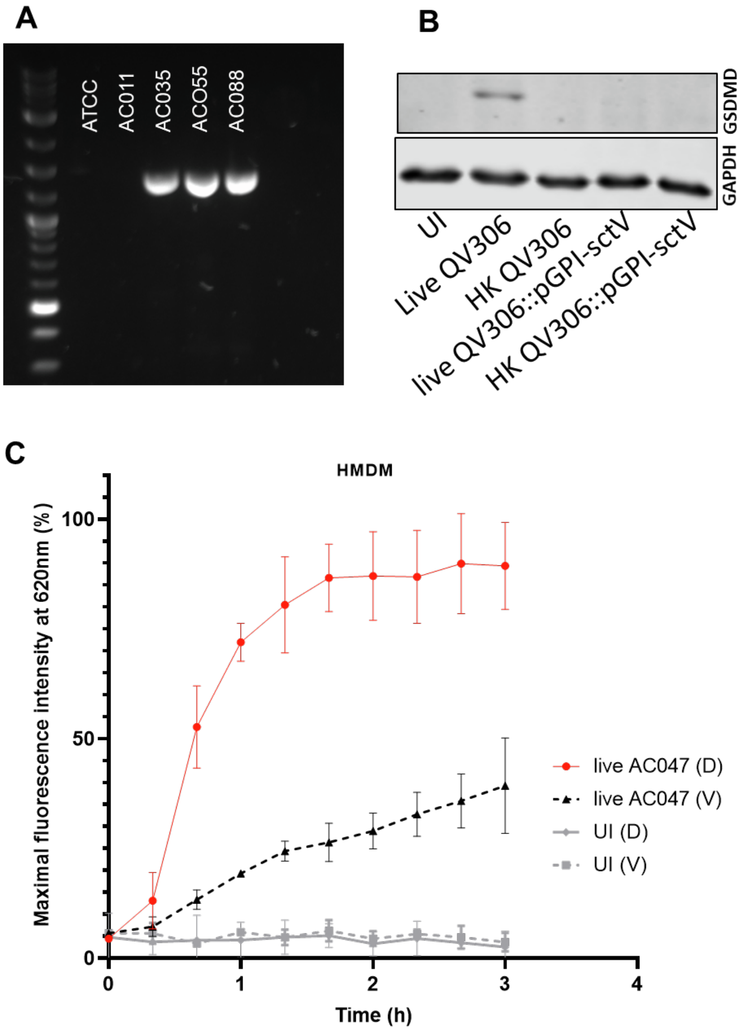

Figure S7. The T3SS is involved in caspase-1-mediated GSDMD cleavage. A. PCR amplification of *sctN* (a conserved T3SS component) in *Achromobacter* clinical isolates. **B.** GSDMD cleavage in HMDMs infected with live and heat-killed (HK) QV306 and mutants at 8-h p.i. (MOI = 20). The anti-GSDMD antibody (CST #36425) detects the cleaved N-terminal domain of GSDMD. **C.** Propidium iodide uptake assay of HMDMs infected with AC047 treated with either 40 µM VX-765 (V) or an equivalent volume of dimethylsulfoxide (D). MOI = 5. Data are represented as mean ± SD from two biological replicates each with 4 technical replicates.

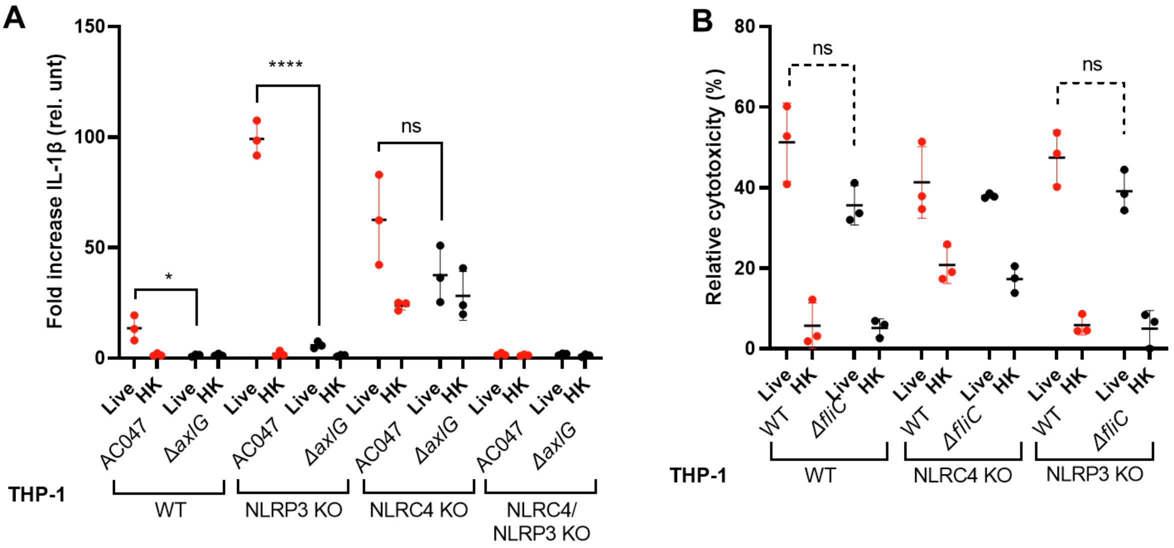

Figure S8. A. Release of IL-1β from single and double KO THP-1 during infection with WT or Δ*axlG* AC047. **B.** LDH release of THP-1 macrophages infected with WT or Δ*fliC* AC047. Data in panels A and B were collected at 5-h p.i. (MOI 20). HK, heat-killed bacteria. Data are represented as mean ± SD from three biological replicates. ****, p < 0.0001 by pairwise t-test analysis.

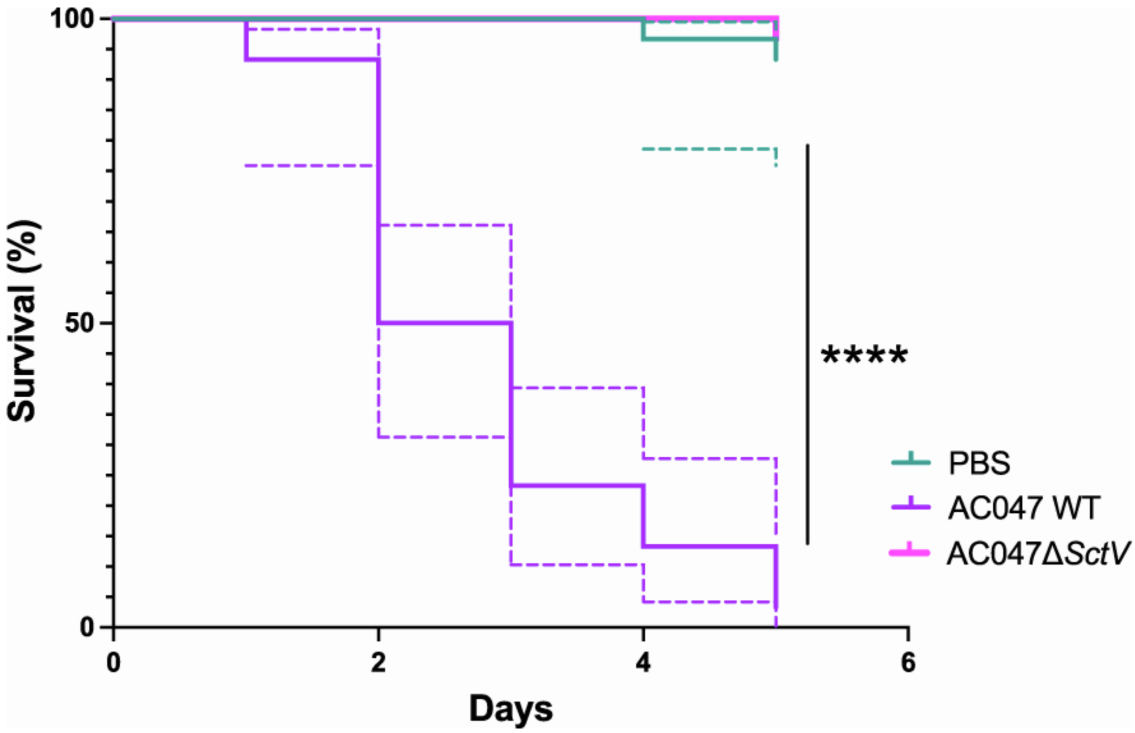

Figure S9. AC047 requires a functional T3SS to efficiently kill *Galleria mellonella* larvae. Larvae were injected with 10^6 CFU (10 µl); PBS was used as a control. Data are represented as mean ± SD from three biological replicates, each with 10 waxworms per test condition. ****, p < 0.0001 by Mantel-Cox Log Rank test.

Table S1. Additional oligonucleotides used in this work[1]

Oligonucleotide [2, 3]	Application	Identifier [4]
5'-cgtgctgacctgacctgagcGCCACATCATCGAGACCTTC	*A. insuavis sctV* mutagenesis plasmid	*A. insuavis sctV* URF
5'-gatgttgtagCCGATGGTCAGGATGGAG	*A. insuavis sctV* mutagenesis plasmid	*A. insuavis sctV* URR
5'-tgaccatcggCTACAACATCCTGCTGTCCGAAG	*A. insuavis sctV* mutagenesis plasmid	*A. insuavis sctV* DRF
5'-cgacggatcccaagcttcttAGCAGGTAAGCGGGCAGG	*A. insuavis sctV* mutagenesis plasmid	*A. insuavis sctV* DRR
5'-CACCATCGACATGGACGAGGCG	*A. insuavis sctV* for Sanger sequencing	*A. insuavis sctV* F
5'-TGCAGGTTCTGTTCCGTGTCGC	*A. insuavis sctV* for Sanger sequencing	*A. insuavis sctV* R
5'-cgtgctgacctgacctgagcCATGCAGGCCAAGCTCAAC	*A. insuavis axlG* mutagenesis plasmid	*A. insuavis axlG* URF
5'-cttctagacggtaccgcatgAGCGGGTCGAGCAGCGCG	*A. insuavis axlG* mutagenesis plasmid	*A. insuavis axlG* DRR
5'-gtgcgcggacCCATTGCCGGTCTCCGGA	*A. insuavis axlG* mutagenesis plasmid	*A. insuavis axlG* URR
5'-ccggcaatggGTCCGCGCACGATGGTCTGAC	*A. insuavis axlG* mutagenesis plasmid	*A. insuavis axlG* DRF
5'-ATCGAGGAAGTGCAGAAGC	*A. insuavis axlG* for Sanger sequencing	*A. insuavis axlG* F
5'-TTCCAGCAGGCATTGGG	*A. insuavis axlG* for Sanger sequencing	*A. insuavis axlG* R
5'-gaaggattcggaattctacaATGGCCGACACCCAAGACC	*A. insuavis axlG* for complementation	*A. insuavis axlG* GCF
5'-cgtcgtcgtccttgtagtctACCATCGTGCGCGGACG	*A. insuavis axlG* for complementation	*A. insuavis axlG* GCR
5'-cgtgctgacctgacctgagcACCATTCGCATCTGGCGG	*A. insuavis sctX* mutagenesis plasmid	*A. insuavis sctX* URF
5'-gttcatccctGCGCCAGTTCCTGGCGTT	*A. insuavis sctX* mutagenesis plasmid	*A. insuavis sctX* URR
5'-gaactggcgcAGGGATGAACGCCTCCATGCCC	*A. insuavis sctX* mutagenesis plasmid	*A. insuavis sctX* DRF
5'-cttctagacggtaccgcatgCGGGGCGCAGGGCAACGT	*A. insuavis sctX* mutagenesis plasmid	*A. insuavis sctX* DRR
5'-ACGAAGTGCTGGTCTACG	*A. insuavis sctX* for Sanger sequencing	*A. insuavis sctX* F
5'-CATCATGAAGATGATCGAGACG	*A. insuavis sctX* for Sanger sequencing	*A. insuavis sctX* R
5'-gaaggattcggaattctacaATGTCCGATATGCGCATCAG	*A. insuavis sctX* for complementation	*A. insuavis sctX* GCF
5'-tgcatgcctgcaggtcgactTCATCCCTGGTACAGCGC	*A. insuavis sctX* for complementation	*A. insuavis sctX* GCR
5'- cagcgcccagCAAGTAGTTGGTGTTAATGACTGC	*A. insuavis fliC* mutagenesis plasmid	*A. insuavis fliC* URR
5'-cgtgctgacctgacctgagcACGCCCTGCACCAATACC	*A. insuavis fliC* mutagenesis plasmid	*A. insuavis fliC* URF
5'- caactacttgCTGGGCGCTGTGCAGAAC	*A. insuavis fliC* mutagenesis plasmid	*A. insuavis fliC* DRF
5'- cttctagacggtaccgcatgATGACTGGGGGCCAGGCG	*A. insuavis fliC* mutagenesis plasmid	*A. insuavis fliC* DRR
5'-GGCCGACGCCCTGGCCAACG	*A. insuavis fliC* for Sanger sequencing	*A. insuavis fliC* F
5'-CGTCAAGTCAGGCGTGCCCTGC	*A. insuavis fliC* for Sanger sequencing	*A. insuavis fliC* R
5'-TTGGTCGGTACCGTCCGGGTGATCCTTGTCCTGG	*A. insuavis hcp* mutagenesis plasmid	*A. insuavis hcp* URR
5'-AACCAGGGTACCTGGAGCCTGACCAAGAACG	*A. insuavis hcp* mutagenesis plasmid	*A. insuavis hcp* DRF
5'-AGACGCGCATGCGCGCAGTCGGTGCAGAAGG	*A. insuavis hcp* mutagenesis plasmid	*A. insuavis hcp* URF
5'-AGACGCTCTAGAGGTCGCGTCGAACGTCACC	*A. insuavis hcp* mutagenesis plasmid	*A. insuavis hcp* DRR
5'-TGGTCGAGAACCTGCCGA	*A. insuavis hcp* for Sanger sequencing	*A. insuavis hcp* F
5'-TGACCGAATATTGCAGCG	*A. insuavis hcp* for Sanger sequencing	*A. insuavis hcp* R
5'-cgtgctgacctgacctgagcACGCGGCGACAAGTTCGTG	*A. xylosoxidans AxoU* mutagenesis plasmid	*A. xylosoxidans AxoU* MSF
5'-cttctagacggtaccgcatgGGGCGAGTTGCCGGTCGT	*A. xylosoxidans AxoU* mutagenesis plasmid	*A. xylosoxidans AxoU* MSR
5'-CCAGTTCGATCTGCTGTTCGACGACC	*A. xylosoxidans AxoU* for Sanger sequencing	*A. xylosoxidans AxoU* F
5'-GCGGAAGACCTGCTGCGTTCG	*A. xylosoxidans AxoU* for Sanger sequencing	*A. xylosoxidans AxoU* R
5'-TGCTGGCGCGGCCGCGGCCACATCATCGAGACCTTCGG	*A. xylosoxidans sctV* mutagenesis plasmid	*A. xylosoxidans sctV* URF
5'-CGTCGCAGCATGCGCCGTCGCCGATGGTCAGGAT	*A. xylosoxidans sctV* mutagenesis plasmid	*A. xylosoxidans sctV* URR
5'-GCTGGAGGCATGCCTACAACATCCTGCTGTCCGA	*A. xylosoxidans sctV* mutagenesis plasmid	*A. xylosoxidans sctV* DRF
5'-GCCACCATCTAGACGCGCTTGATGTTGTCCACCAGCTT	*A. xylosoxidans sctV* mutagenesis plasmid	*A. xylosoxidans sctV* DRR
5'-CACCATCGACATGGACGAGGCG	*A. xylosoxidans sctV* for Sanger sequencing	*A. xylosoxidans sctV* F
5'-TGCAGGTTCTGTTCCGTGTCGC	*A. xylosoxidans sctV* for Sanger sequencing	*A. xylosoxidans sctV* R

[1] All oligonucleotide primers were provided by Eurofins Scientific.
[2] Bases in lower case denotes vector overlap sequence for Gibson assembly
[3] Underlined bases indicate enzyme restriction sites for cloning
[4] Abbreviations: URF, upstream region forward; URR, upstream region reverse; DRF, downstream region forward; DRR, downstream region reverse; F, forward; R, reverse; GCF, gene cloning forward; GCR, gene cloning reverse; MSF, middle section forward; MSR, middle section reverse.
